# Childhood adversity and DNA methylation in two population-based cohorts

**DOI:** 10.1038/s41398-018-0307-3

**Published:** 2018-12-03

**Authors:** L. C. Houtepen, R. Hardy, J. Maddock, D. Kuh, E. L. Anderson, C. L. Relton, M. J. Suderman, L. D. Howe

**Affiliations:** 1 MRC Integrative Epidemiology Unit at the University of Bristol, Population Health Sciences, Bristol Medical School, University of Bristol, Bristol, UK; 2 MRC Unit for Lifelong Health and Ageing, University College London, London, UK

## Abstract

Childhood adversity affects later health, but the underlying molecular mechanisms are unclear. Although there is some evidence from animal models and case-control studies of a role for DNA methylation, evidence from human population-based studies is limited. In two cohorts (mothers from the Avon Longitudinal Study of Parents and Children, ALSPAC, *n* = 780 and women from the MRC National Survey of Health and Development, NSHD, *n* = 552), we assessed the association of seven adverse childhood experiences (ACEs: parental physical illness, parental mental illness, parental death, parental separation, suboptimal maternal bonding, childhood illness and child maltreatment) as well as their combination (ACE score) with genome-wide DNA methylation levels measured using the Illumina Infinium HumanMethylation450 BeadChip in peripheral blood at mean age 47 years (ALSPAC) and in buccal cells at age 53 years (NSHD). CpG sites with a genome-wide false discovery rate (FDR) below 0.05 and differentially methylated regions (DMRs) with one-step Šidák correction *p*-values below 0.05 in each cohort were examined in the other cohort. No individual CpG sites replicated across cohorts. However, nine DMRs replicated across cohorts respectively associated with the ACE score (one region), parental mental illness (two regions), parental physical illness (three regions) and parental death (three regions). These observations indicate that some adverse childhood experiences, notably those related to parental health, may leave imprints on peripheral DNA methylation that persist to mid-life.

## Introduction

Childhood adversity is related to a broad range of negative outcomes across the lifespan including poorer mental and physical health^1,2^ as well as lower educational attainment, income and economic participation^3,4^. The molecular mechanisms underlying these associations remain uncertain, although a role for epigenetic marks has been suggested. DNA methylation, the addition of a methyl group to a cytosine base that is followed by a guanine (CpG site)^5^, is the epigenetic modification most widely analysed in population-based studies. Previous studies indicate DNA methylation in adulthood can be affected by environmental factors early in life^6,7^.

Childhood adversity is related to altered DNA methylation in both animal and human studies (see review^8^). In fact, several genome-wide studies of childhood adversity in humans have identified associations throughout the genome^6,9–15^. Moreover, there is some evidence that associations near stress-related genes may be relevant for later life health^16,17^. However, most previous studies either focused on candidate genes or sampled from at-risk populations. One recent genome-wide population-based study on early-life victimisation stress did not identify robust changes in DNA methylation^18^, but did not examine other forms of childhood adversity (parental separation, parental mental or physical illness, child illness).

Therefore, we examine the association between various types of childhood adversity and genome-wide DNA methylation in two large population-based cohorts, the mothers of the Avon Longitudinal Study of Parents and Children (ALSPAC) and women from the MRC National Survey of Health and Development (NSHD). In these cohorts, previous studies have demonstrated associations between adversity and health^19–23^. Given the known co-occurrence of different types of adversity and the potential cumulative effects on health^24,25^, we analyse a count score of the adverse childhood experiences (ACE) in addition to exploring whether each different type of childhood adverse experience was associated with DNA methylation variation in separate epigenome-wide analyses (EWAS).

## Materials and methods

### Data

The ALSPAC is a prospective, population-based birth cohort study that recruited 14,541 pregnant women resident in Avon, UK, with expected delivery dates between the 1st April 1991 and 31st December 1992^26,27^. The mothers, their partners and the index child have been followed-up through clinics, questionnaires and links to routine data. The ALSPAC mothers form the participants for this study. The study website contains details of all the data that is available through a fully searchable data dictionary (http://www.bris.ac.uk/alspac/researchers/data-access/data-dictionary/). The Accessible Resource for Integrated Epigenomic Studies (ARIES) project^28^ includes 1018 mother-child pairs from ALSPAC that had DNA methylation measured using the Infinium HumanMethylation450BeadChip (Illumina, Inc). Our analysis used DNA methylation profiles of peripheral blood collected from mothers at a follow-up clinic (age of participants ranged from 44 to 50 years (mean = 47.1)). DNA methylation profiles derived from peripheral blood collected during the study pregnancy approximately 18 years earlier were used to replicate findings (mean age = 29.1). Ethical approval was obtained from the ALSPAC Ethics and Law Committee and the Local Research Ethics Committees and informed consent was obtained from all subjects.

The MRC National Survey of Health and Development (NSHD) is based on a nationally representative sample of 5362 births out of all the single births that took place within marriage in one week in March 1946 in England, Scotland and Wales;^29^ study members have been followed up 24 times, up to age 69^30^.

For 810 women, DNA methylation at age 53 years was measured using the Infinium HumanMethylation450BeadChip (Illumina, Inc) on buccal epithelial samples and in a subsample of 156 women also on whole blood cells (WBC)^31^. All women gave written informed consent for their samples to be used in genetic studies of health, and the Central Manchester Ethics Committee approved the use of these samples for epigenetic studies of health in 2012.

### Childhood adversity

Seven ACEs were assessed in both ALSPAC and NSHD: (i) suboptimal maternal bonding, (ii) childhood physical illness, (iii) parental mental illness, (iv) parental divorce or separation, (v) parental physical illness or disability, (vi) parental death in childhood and (vii) child maltreatment. In ALSPAC child maltreatment was further refined into five distinct ACEs, three forms of abuse (sexual, emotional and physical) and two forms of neglect (emotional and physical). In ALSPAC, women reported ACEs in retrospective questionnaires at study enrolment (during pregnancy, mean age 28 years) or soon thereafter. In NSHD, ACEs were prospectively reported in interviews and questionnaires by the participants’ mothers up to age 16, except for parental bonding and maltreatment, which were self-reported when participants were age 43. All ACEs were defined as binary indicators of presence/absence of the adversity. An ACE score was generated by adding up whether an individual was exposed to the ACEs, creating a score with a maximum possible value of seven in NSHD and eleven in ALSPAC (further details in Supplementary information).

Associations between the ACE score and mental health have been demonstrated previously in the NSHD cohort^23^, but not in the ALSPAC mothers. Therefore, to demonstrate that the ACE score behaves as expected in relation to mental health, we examined the association of the ACE score with depression (defined according to a score of 13 or more on the Edinburgh Postnatal Depression Score from questionnaires completed by the mother when the child was 11 years old), both in the full ALSPAC cohort and in the subsample included in our EWAS.

### DNA methylation

In both cohorts following DNA extraction, DNA was bisulfite converted using the Zymo EZ DNA MethylationTM kit (Zymo, Irvine, CA, USA). Genome-wide DNA methylation levels at over 485,000 CpG sites were measured with the Infinium HumanMethylation450 BeadChip. Both datasets were pre-processed in R version 3.2.4 with the meffil package^32^, using functional normalisation^33^ to reduce non-biological differences between probes. After pre-processing, 976 samples and 453,965 probes were available for further analysis in ALSPAC, while 766 buccal as well as 153 blood samples and 455,971 probes were available for further analysis in NSHD (further details in Supplementary information). Methylation level at a CpG site is expressed as a ‘beta’ value (β-value), ranging from 0 (no cytosine methylation) to 1 (complete cytosine methylation). β-Values are reported as percentages. To reduce the influence of outliers in regression models, normalized β-values were 90%-Winsorized.

### Epigenome-wide association studies (EWAS)

Epigenome-wide association studies (EWAS) for the ACE score and each separate ACE were conducted in R version 3.2.4^34^, using linear regression models with untransformed methylation beta values as the outcome. EWAS were conducted separately in each cohort; 8 separate EWAS were performed in NSHD (7 individual ACEs and the ACE score), and 13 EWAS were performed in ALSPAC (11 individual ACEs, child maltreatment, and the ACE score). Multiple testing was accounted for by controlling the false discovery rate (FDR) at 5%, implementing the method by Benjamini and Hochberg^35^.

All models were adjusted for the age at DNA sampling. To adjust for technical batch effects, independent surrogate variables (ISV) were calculated and included in all models^36^. In ALSPAC, Houseman-estimated cell proportions^37^ were used to adjust for cellular heterogeneity in blood DNA samples.

As smoking may potentially lie on the causal pathway between childhood adversity exposure and DNA methylation, adjustment would remove part of the pathway of interest and could potentially result in collider bias. Thus, the main analyses were not adjusted for smoking status. However, the influence of smoking for the CpGs and DMRs of interest was examined in a sensitivity analysis by adding a methylation-based smoking score, derived from the effect size estimates of the 185 probes with FDR <0.05 in a recent smoking EWAS^38^, as a covariate to the main model.

When the ACE score was the exposure, the regression coefficients in the EWAS model are interpreted as the difference in mean methylation when the score increases by one. For the individual ACEs, regression coefficients are interpreted as the mean difference in methylation between the adversity exposed and unexposed groups.

### Differentially methylated regions

The EWAS results were used to detect differential methylation across larger regions of the genome with Comb-P^39^ (further details in Supplementary information).

### Replication

CpGs with FDR <0.05 or DMRs with Šidák-corrected *P*-value <0.05 in either ALSPAC or NSHD were examined in the other cohort.

### Enrichment for probes previously reported in literature

To examine whether the top 1000 CpGs in our EWAS analyses were enriched for CpGs previously reported in literature, we identified nine publications that reported CpG sites on the Illumina Infinium HumanMethylation450 BeadChip associated with (a form of) childhood adversity (further details in Supplementary information, Supplementary Table 5 and the probe lists in Supplementary data sheet 1). We also included lists for potentially unreliable probes^40,41^ and CpGs related to smoking^38^. For all lists, enrichment of the previously reported CpGs in the top 1000 CpGs in our EWAS analyses was examined using Fisher’s exact test.

### Cohort comparison

We compared the associations of adversity in ALSPAC and NSHD by calculating Pearson’s correlation of the regression coefficients of the top 1000 CpG sites identified by each cohort. For DMRs, correlation was calculated from the average of the regression coefficients of the CpGs that were part of a DMR. To calculate an informative correlation coefficient, the cohort comparison was only performed if, for a particular ACE, more than three DMRs were identified.

### Cross-tissue analysis

In a subset of 98 women in NSHD with buccal DNA and ACE data, who also had blood DNA methylation measured at the same time point, the tissue specificity of childhood adversity related changes in DNA methylation was examined (cross-tissue subset). Parental mental illness could not be evaluated in this subsample due to a lack of cases (see Supplementary Table 4). For each of the available ACE measures, an EWAS analysis was performed for each tissue type. We then examined the tissue specificity of adversity by calculating the correlation of the regression coefficients for the top 1000 CpGs. For DMRs, we calculated the correlation of the average of the regression coefficients of the CpGs that were part of each DMR. The cross-tissue analysis was only performed if, for a particular ACE, more than three DMRs were identified.

### Genetic variants near replicated DMRs

In a sensitivity analysis, the influence of genetic variation on the association between childhood adversity and methylation at our DMRs of interest was investigated in ALSPAC. Genetic data from genome-wide assays (Illumina 660W-quad BeadChip) was available for 700 of the 780 participants in the ALSPAC EWAS analyses^26^.

For each replicated DMR, we included SNPs as covariates in the EWAS model that were located within 1MB (observed and imputed SNPs pruned for independence (R-squared <0.0001) with a major allele frequency >1% and less than 3% missing) and were associated (*p* < 0.05) with at least one CpG in the DMR.

## Results

### Characteristics of participants

Complete adversity and DNA methylation data were available for 780 women in the ALSPAC cohort and 552 women in NSHD. The mean age at DNA methylation assessment was 47.4 years in ALSPAC, and 53.4 years in NSHD. Table 1 shows the prevalence of each type of ACE, and each category of the ACE score. Parental physical illness and parental death had similar prevalence in both cohorts. Parental mental illness, parental separation and any maltreatment were more prevalent in ALSPAC, and child illness and suboptimal maternal bonding were more common in NSHD. Most ACEs had a similar prevalence in the participants without DNA methylation data, and the ACE score demonstrated similar associations with depression in the full cohort of ALSPAC mothers and in the subsample included in our analysis (see Supplementary Tables 2–4).

**Table 1:** Prevalence of childhood adverse experiences in ALSPAC mothers and NSHD participants

	ALSPAC		NSHD		Comparison^a^
	Participants with complete adversity data (*n* = 8021)	Participants with DNA methylation data, included in our analysis (*n* = 780)	Participants with complete adversity data (*n* = 2438)	Participants with DNA methylation data, included in our analysis (*n* = 552)	*p*-value
Sex = Male (%)	0	0	51.4	0	n/a
Age clinic (mean (sd))	47.90 (4.40)	47.14 (4.61)	53.48 (0.50)	53.45 (0.50)	<0.001
Smoking (%)					<0.001
Never-smoker	53.4	56.3	30.3	33.3	
Ex-smoker	35.4	36.2	48.5	47.3	
Current-smoker	11.1	7.4	21.2	19.4	
Childhood adverse experiences before 17 years					
Parent physically ill (%)	25.8	28.6	22.7	25.9	0.308
Parent mentally ill (%)	4.4	5.8	2.2	1.8	0.001
Parents separated (%)	17.7	14.9	5	4.2	<0.001
Parent died (%)	5.9	5.1	6.9	6.3	0.409
Suboptimal maternal bonding (%)	18.8	15.8	19.3	21.7	0.007
Child illness (%)	5	5.3	15.4	13.4	<0.001
Child maltreatment (%)	24.6	22.2	6.2	6.5	<0.001
Sexual abuse (%)	4.5	3.7	n/a	n/a	n/a
Physical abuse (%)	3.2	3.1	n/a	n/a	n/a
Emotional abuse (%)	7.5	8.3	n/a	n/a	n/a
Physical neglect (%)	1.8	1.2	n/a	n/a	n/a
Emotional neglect (%)	20.8	19.4	n/a	n/a	n/a
Adverse Childhood Experiences (ACE) count score (%)					<0.001
0	42.4	43.8	45.3	42	
1	29	29.4	36.8	41.1	
2	14	12.3	13.7	12.7	
3	7.4	6.9	3.6	3.4	
4	3.5	4	0.6	0.5	
5	1.9	1.9	0.1	0.2	
6	1	0.9	0	0	
7	0.5	0.8	0	0	
8	0.2	0	n/a	n/a	
9	0.1	0	n/a	n/a	
10	0	0	n/a	n/a	

^a^The difference between the ALSPAC (*n* = 780) and NSHD (*n* = 552) participants that were included in our EWAS analyses was tested using a Pearson’s chi-squared test for categorical variables and an ANOVA for numerical variables

The correlation (Cramér’s V for nominal variables) between different adverse experiences varied, with ϕ_c_ ranging from 0 to 0.5 in ALSPAC and 0 to 0.1 in NSHD (Supplementary Figure 3 and 4). In general, correlations between ACEs were considerably lower in NSHD than ALSPAC.

### Individual CpGs

In both NSHD and ALSPAC, there was no association between the cumulative ACE score and individual CpG sites after multiple testing. In NSHD, DNA methylation levels at three CpG sites were negatively associated (FDR < 0.05) with a specific adversity; parental mental illness (cg17164016 *B* = −0.07, *p* = 8.8 × 10^–08^, FDR = 0.043), childhood illness (cg10303653 *B* = −0.017, *p* = 2.1 × 10^–08^, FDR = 0.010) and child maltreatment (cg14296561 *B* = −0.040, *p* = 6.5 ×^–09^, FDR = 0.003) (see Table 2). Similar associations were found after adjusting for smoking in the EWAS model (cg17164016 *B* = −0.07, *p* = 2.5 × 10^–07^, FDR = 0.12; cg10303653 *B* = −0.016, *p* = 2.2 × 10^–07^, FDR = 0.11 and cg14296561 *B* = −0.043, *p* = 8.0 × 10^–10^, FDR = 0.0004). None of these associations were replicated in ALSPAC, although cg14296561 was weakly but positively (opposite direction of effect compared with NSHD) associated with child maltreatment (B = 0.0091, unadjusted *p* = 0.041) (see Table 2).

**Table 2:** CpGs surviving FDR correction in an epigenome-wide analysis (EWAS)

Discovery	ProbeID	Gene	Position	*B*	*p*	fdr	Look up NSHD	Look up ALSPAC
NSHD-Parent mentally ill	cg17164016		chr8: 110990365	−0.067	8.86E−08	0.043	ACE score *B* = -0.0026, *p* = 0.20 Parent physically ill *B* = −0.0054, *p* = 0.16 **Parent mentally ill** ***B*** = −0.067, *p* = 8.9e−08^*^ Parents separated *B* = -0.00068, *p* = 0.94 Parent died *B* = −0.019, *p* = 0.006^*^ Suboptimal maternal bonding B = 0.0061, *p* = 0.14 Child illness *B* = 0.0012, *p* = 0.81 Child maltreatment *B* = 0.0052, *p* = 0.46	ACE score *B* = 0.00093, *p* = 0.33 Parent physically ill *B* = -0.0018, *p* = 0.56 **Parent mentally ill** ***B*** = 0.0039, *p* = **0.49** Parents separated *B* = −0.0014, *p* = 0.72 Parent died *B* = 0.0075, *p* = 0.22 Suboptimal maternal bonding *B* = 0.0016, *p* = 0.65 Child illness *B* = 0.0073, *p* = 0.23 Child maltreatment *B* = 0.0035, *p* = 0.28 Sexual abuse *B* = 0.0044, *p* = 0.53 Physical abuse *B* = 0.0077, *p* = 0.33 Emotional abuse *B* = −0.00036, *p* = 0.94 Physical neglect *B* = 0.0049, *p* = 0.70 Emotional neglect *B* = 0.0035, *p* = 0.29
NSHD-Child illness	cg10303653	*TNXB*	chr6: 32049516	−0.017	2.10E−08	0.010	ACE score *B* = −0.0032, *p* = 0.012^*^ Parent physically ill B = −0.0013, *p* = 0.59 Parent mentally ill B = 0.0057, *p* = 0.47 Parents separated B = −0.0033, *p* = 0.54 Parent died B = 0.0022, *p* = 0.63 Suboptimal maternal bonding B = 0.00074, *p* = 0.78 **Child illness** ***B*** = −0.017, *p* = 2.1e−08^*^ Child maltreatment *B* = −0.00091, *p* = 0.84	ACE score *B* = 0.00034, *p* = 0.76 Parent physically ill *B* = 0.0021, *p* = 0.54 Parent mentally ill *B* = −0.0077, *p* = 0.24 Parents separated *B* = 0.0087, *p* = 0.054 Parent died *B* = −0.0066, *p* = 0.34 Suboptimal maternal bonding *B* = −0.00064, *p* = 0.88 **Child illness** ***B*** = 0.0057, *p* = **0.41** Child maltreatment *B* = 0.001, *p* = 0.78 Sexual abuse *B* = 0.003, *p* = 0.71 Physical abuse *B* = -0.0023, *p* = 0.8 Emotional abuse *B* = 0.00043, *p* = 0.94 Physical neglect *B* = −0.015, *p* = 0.31 Emotional neglect *B* = −0.00034, *p* = 0.93
NSHD-Child maltreatment	cg14296561	*BIRC3*	chr11: 102203542	−0.040	6.51E−09	0.003	ACE score *B* = −0.0083, *p* = 4.4e−05^*^ Parent physically ill *B* = −0.0062, *p* = 0.12 Parent mentally ill *B* = −0.0058, *p* = 0.66 Parents separated *B* = −0.0033, *p* = 0.7 0 Parent died *B* = −0.0072, *p* = 0.32 Suboptimal maternal bonding B = −0.0041, *p* = 0.33 Child illness *B* = −0.0094, *p* = 0.061 **Child maltreatment** ***B*** = -0.04, *p* = 6.5e−09^*^	ACE score *B* = 0.0017, *p* = 0.20 Parent physically ill *B* = 0.0025, *p* = 0.55 Parent mentally ill *B* = −0.0019, *p* = 0.81 Parents separated *B* = 0.012, *p* = 0.026^*^ Parent died *B* = −0.0028, *p* = 0.74 Suboptimal maternal bonding *B* = −0.0028, *p* = 0.59 Child illness *B* = 0.0043, *p* = 0.61 **Child maltreatment** ***B*** = 0.0091, *p* = 0.041^*^ Sexual abuse *B* = 0.016, *p* = 0.1 0 Physical abuse *B* = 0.0017, *p* = 0.87 Emotional abuse *B* = 0.013, *p* = 0.048^*^ Physical neglect *B* = −0.02, *p* = 0.26 Emotional neglect *B* = 0.0055, *p* = 0.25

*In the last two columns indicates a nominal significant association between the CpG and one of the childhood adversities in the respective cohort, NSHD or ALSPAC.

Bold in the final two columns represents the ACE to which the discovery analysis relates.

In ALSPAC, there was no associations of individual CpG sites with any ACE measure after adjustment for multiple tests (FDR < 0.05) (see Supplementary Figure 5 and 6 for QQ-plots of the EWAS analyses in NSHD and ALSPAC, respectively).

### Differentially methylated regions

We identified 231 differentially methylated regions (DMRs; one-step Šidák correction <0.05) associated with specific ACE measures in ALSPAC (*n* = 97) and NSHD (*n* = 134). Four of the 134 NSHD DMRs replicated in ALSPAC and six of the 97 ALSPAC DMRs replicated in NSHD (same ACE measure and direction of effect; StoufferLiptak-Kechris corrected *P*-value <0.05 unadjusted for genome-wide tests), even after adjusting for smoking (see Table 3 and Supplementary Table 7).

**Table 3:** DMRs that replicated across cohorts

ACE	DMR	Nr CpGs	Gene	ALSPAC (Direction^a^, *p*-value)	NSHD (Direction^a^, *p*-value)	Replicated ACEs in lookup^b^
ACE count score	chr8: 145654565-145654855	5	*VPS28, TONSL*	----- *p* = 7.5e−08*	----- *p* = 1.8e−06*	ACE count score, Parent died, Maternal bond, Child maltreatment
Parent mentally ill	chr12: 14720726-14721289	10	*PLBD1*	-++-++++++ *p* = 0.0058	+++++++++- *p* = 9.5e−07*	Parent mentally ill
Parent mentally ill	chr1: 3104999-3105327	5	*PRDM16*	----- *p* = 0.001	----- *p* = 7.4e−08*	Parent mentally ill
Parent physically ill	chr15: 81426347-81426670	9	*C15orf26*	+++++++++ *p* = 0.011	+++++++++ *p* = 7.4e–07*	Parent mentally ill
Parent physically ill	chr22: 27834439-27834630	3	n/a	--- *p* = 6.9e−06*	--- *p* = 0.045	Parent mentally ill
Parent physically ill	chr8: 144120335-144120707	7	*C8orf31*	+++++++ *p* = 4.4e−06*	+++++++ *p* = 0.00092	Parent mentally ill, Parent died
Parent died	chr15: 40364524-40364863	3	n/a	+++ *p* = 6.3e−07*	+++ *p* = 0.0044	Parent died, Parent mentally ill
Parent died	chr7: 24323261-24323940	9	*NPY*	+++++++++ *p* = 9.2e−07*	+++++++++ *p* = 0.014	Parent died
Parent died	chr2: 18766018-18766295	4	*NT5C1B*	--- + *p* = 9.8e−06*	---- *p* = 0.014	Parent died

^a^Direction of effect for each individual CpG that is part of the DMR was derived from the regression coefficient in the epigenome-wide analysis for individual CpGs

^b^If the DMR additionally replicated for another ACE (same direction of effect and StoufferLiptak-Kechris corrected *P*-value <0.05 in both ALSPAC and NSHD), the additional ACE is mentioned in this column

*DMR passed Šidák correction for multiple testing in comb-p

Of the nine replicated DMRs, the one (chr8:145654565-145654855) DMR negatively associated with the ACE score survived correction for multiple testing (Šidák corrected *p*-value <0.05) and was thus discovered independently in both cohorts, two DMRs were positively (chr12:14720726-14721289) or negatively (chr1:3104999-3105327) associated with parental mental illness, three DMRs were either positively (chr15:40364524-40364863 and chr7:24323261-24323940) or negatively (chr2:18766018-18766295) associated with parental death and the last three DMRs were either positively (chr15:81426347-81426670 and chr8:144120335-144120707) or negatively (chr22:27834439-27834630) associated with parental physical illness.

Only two of the eight DMRs associated with a specific ACE were additionally associated with another ACE in both cohorts (chr15:40364524-40364863 with parental mental illness; chr8:144120335-144120707 with parental death). In contrast, the DMR (chr8:145654565-145654855) associated with the ACE score, was also related to three specific ACEs (parental death, suboptimal maternal bond and child maltreatment) in both cohorts (see Table 3).

Besides the nine replicated DMRs, nineteen DMRs were associated with the abuse and neglect childhood adversity measures that were solely available in ALSPAC (3 DMRs emotional abuse, 5 DMRs emotional neglect, 8 DMRs physical abuse, 2 DMRs physical neglect and 1 DMR sexual abuse) even after adjusting for smoking (see Supplementary Table 8). Although these nineteen DMRs could not be replicated in NSHD, the DMR on chr3:87138203-87138701 was positively associated with emotional neglect in ALSPAC as well as child maltreatment in both ALSPAC and NSHD. Finally, the DMR on chr11:67417958-67418406 was negatively associated with several abuse and neglect measures in ALSPAC (emotional neglect, emotional abuse and child maltreatment) but did not replicate in NSHD where the DMR was positively associated with child maltreatment.

Since there are DNA methylation profiles for many of the same ALSPAC participants measured about 18 years earlier during pregnancy (*n* = 769), we tested the 9 replicated DMRs for replication at that earlier time point. Five of the 9 were replicated, including the single DMR that survived genome-wide adjustment for multiple testing in both cohorts (chr8:145654565-145654855). Details are provided in Supplementary Table 9.

### Enrichment for probes previously reported in literature

Although some of our top 1000 CpGs were enriched for certain previously reported CpGs (1 CpG^18^, 4 CpGs^11^, 2 CpGs^6^, 2 CpGs^15^ and 2 CpGs^10^) (Supplementary Figure 7), in most cases the type of adversity in our analysis was very different from the original study. Only three previously reported CpGs replicated (*p* < 0.05) and were amongst the top 1000 CpGs of our analyses: (i) cg19335412 near the *ACTA2* gene that was previously linked to childhood abuse^6^ and to sexual abuse in ALSPAC (*B* = 0.039, *p* = 0.0014), (ii) cg00973947 near the *C3orf58* gene that was previously linked to the childhood trauma questionnaire score^10^ and to ACE score in NSHD (*B* = 0.001, *p* = 0.0014), and (iii) cg27347930 near the *LIG3* and *CDS* genes that was previously linked to sexual abuse in the Dunedin study (*B* = -0.008, *p* = 2.39 × 10^−08^)^18^ and also to sexual abuse in ALSPAC (*B* = −0.016, *p* = 0.004).

There was some evidence for enrichment of smoking-related associations^38^ and potentially unreliable probes^40,41^ in our top CpGs (Supplementary Figure 7).

### Cohort comparison

For all ACEs measured in both cohorts, the regression coefficients of the top 1000 CpGs were only weakly correlated between cohorts (Spearman *ρ* range between −0.12 and 0.08, mean = −0.003, sd = 0.05) (see Supplementary Figure 8). Furthermore, the average regression coefficients of the CpGs that are part of DMRs were also only generally weakly and inconsistently correlated between cohorts (*ρ* range between −0.59 and 0.60, mean = 0.009, sd = 0.29) (see Supplementary Figure 9).

### Cross-tissue analysis

In the NSHD subsample with both blood and buccal cells (*n* = 98), the regression coefficients of the top 1000 CpGs in the buccal EWAS were moderately to highly correlated with the regression coefficients of the same CpGs in blood (Spearman *ρ* range = 0.20–0.59, mean = 0.50, sd = 0.13). Similar moderate to high cross-tissue correlations were present for the top 1000 CpGs in the blood EWAS analyses (ρ range = 0.36–0.69, mean = 0.54, sd = 0.10) (see Supplementary Figure 10). The average regression coefficients of the CpGs that are part of DMRs were also highly correlated across tissue type (*ρ* range = 0.66–0.95, mean = 0.85, sd = 0.12 for blood-based ACE DMRs and ρ range = 0.45–0.86, mean = 0.73, sd = 0.15 for buccal-based ACE DMRs) (see Supplementary Figure 11).

### Influence of genetic variants on replicated DMRs

In ALSPAC, between eight and 40 genetic variants were within 1MB and associated with at least one CpG for one of the nine replicated DMRs (see Table 4). After adding the appropriate genetic variants as covariates to the original EWAS model, the regression coefficients for most CpGs in the DMRs were in the same direction as the original EWAS model and all DMRs were still statistically significant (one-step Šidák correction <0.05).

**Table 4:** Influence of genetic variants near the replicated DMRs in ALSPAC

ACE	DMR	Nr CpGs	Gene	Original analysis (Direction^a^, *p*-value)	Nr SNPs (within 1MB, associated one CpG (*p* < 0.05))	Sample size	Sensitivity analysis, including SNPs (Direction^a^, *p*-value)^b^
ACE count score	chr8: 145654565-145654855	5	*VPS28, TONSL*	----- *p* = 7.5e−08*	8	618	----- *p* = 0.0020
Parent mentally ill	chr12: 14720726-14721289	10	*PLBD1*	-++-++++++ *p* = 0.0058	28	532	--+±++++++ *p* = 0.0496
Parent mentally ill	chr1: 3104999-3105327	5	*PRDM16*	----- *p* = 0.001	16	672	----- *p* = 0.001
Parent physically ill	chr15: 81426347-81426670	9	*C15orf26*	+++++++++*p* = 0.011	20	609	+++++++++*p* = 0.0010
Parent physically ill	chr22: 27834439-27834630	3	n/a	--- *p* = 6.9e−06*	35	686	--- *p* = 1.207e−05*
Parent physically ill	chr8: 144120335-144120707	7	*C8orf31*	+++++++ *p* = 4.4e−06*	13	673	+++++++ *p* = 7.998e−05*
Parent died	chr15: 40364524-40364863	3	n/a	+++ *p* = 6.3e−07*	19	605	+++ *p* = 8.915e−06*
Parent died	chr7: 24323261-24323940	9	*NPY*	+++++++++ *p* = 9.2e−07*	40	584	+++++-+++ *p* = 0.0005
Parent died	chr2: 18766018-18766295	4	*NT5C1B*	--- + *p* = 9.8e−06*	27	520	-± -- *p* = 0.0289

^a^Direction of effect for each individual CpG that is part of the DMR was derived from the regression coefficients for individual CpGs

^b^The CpGs with a different direction of effect in the sensitivity analysis are underlined and in red.

*DMR passed Šidák correction for multiple testing in comb-p

## Discussion

In this epigenome-wide study of childhood adversity in two population-based cohorts of mid-life women, we discovered nine novel differentially methylated genomic regions (DMRs) but no individual CpGs that replicated across cohorts. One DMR was associated with a measure for cumulative adversity (ACE count score), whereas the other eight regions were associated with specific types of adversity, namely parental mental illness (two regions), parental physical illness (three regions) and parental death (three regions).

**Fig. 1:**
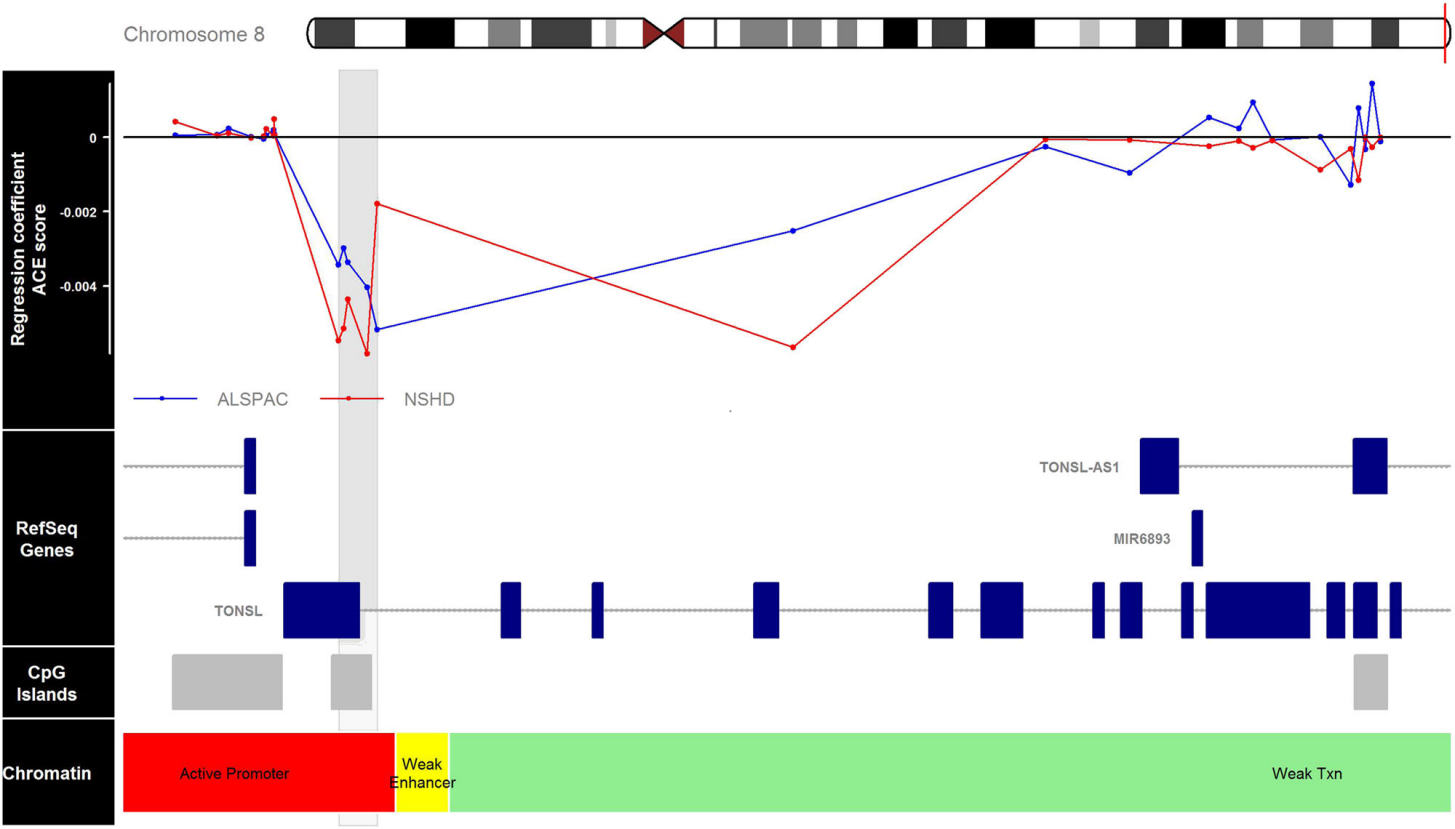
Overview of the area 1600 bp upstream and 8000 bp downstream of the DMR associated with the ACE score in both ALSPAC and NSHD. The DMR is shaded across all panels by a light grey rectangle. The top panel displays the regression coefficient for the association between DNA methylation and the ACE count score in ALSPAC (blue, *n* = 780) and NSHD (red, *n* = 552). The other panels indicate respectively the presence of coding exons (blue blocks) and non-coding introns (grey line) of the genes near this area (second panel), the location of CpG islands (third panel) and chromatic accessibility (fourth panel) based on information extracted for genome build Hg19 from the UCSC website^49^ with the gviz R package^50^. Abbreviations: chr, chromosome

The most robust observation in this study was for a DMR negatively associated with the ACE score, which was also associated with parental death, suboptimal maternal bonding and child maltreatment in ALSPAC and NSHD. The DMR is located near a CpG island of the promotor of the tonsoku-like DNA repair protein (*TONSL*) gene (see Fig. 1), which is a histone chaperone that may counteract chromatin compaction by preventing the association between the histone H4 tail with the H2A–H2B acidic patch on neighbouring nucleosomes^42^. The DMRs related to a specific ACE tended to be related to only one adversity, which is in line with a previous study where different DNA methylation CpG sites were affected by specific types of adversity compared to several types of childhood adversity^11^. This appears to suggest that certain parts of the methylome are associated with distinct adverse childhood experiences, while other locations reflect more general exposure to adversity during childhood.

The eight replicated DMRs related to a specific ACE were all associated with parental health measures (parental mental illness, parental physical illness and parental death). This could indicate that parental health has a bigger impact on the child’s epigenome than other types of adversity. Alternatively, it may reflect intergenerational transmission of health from parent to child with the woman’s own health in turn influencing her epigenome. Parental physical illness and parental death are linked to six of the eight replicated DMRs. These ACEs have the most similar prevalence in ALSPAC and NSHD suggesting that similarity of measurements and hence effective harmonisation across cohorts aided replication. Our lack of associations for other measures of adversity is in line with a recent epigenome-wide study reporting limited evidence for an association between DNA methylation and several forms of early-life victimisation stress^18^.

The replication of DMRs is noteworthy given the considerable differences between ALSPAC and NSHD in tissue type (blood or buccal), source of reporting (retrospective self-report or mother during childhood), and years of birth (1950–1976 and 1946). The cohort differences may explain differences in adversity prevalence which are known to be affected by source of reporting^43–45^. Our own cross-cohort analyses also suggest cohort differences may play a role, as the regression coefficients for the same ACE exposure did not correlate across cohorts but were correlated between tissue types within the same cohort despite large, genome-wide DNA methylation differences between tissues^46^. Furthermore, our genetic analysis does not indicate the methylation differences at the DMRs are driven solely by genetic variation, as the direction of effect for the individual CpGs is similar and the DMRs remain significant after adjusting for genetic variants within 1MB of the DMR.

Our tissue comparison showed medium to high correlation across buccal and blood tissue for CpGs and DMRs associated with the same ACE, whereas the cross-tissue correlation was lower for different ACEs. This suggests similar childhood adversity related DNA methylation changes could be identified in blood and buccal tissue. Some recommend buccal samples for population epigenetic studies, as they contain more hypomethylated DNA regions, which tend to cluster around disease-associated SNPs;^47^ others, however, argue that demographic factors may be better reflected in blood DNA methylation patterns^46^. Identifying DMRs that are associated in both tissue types supports the robustness of our findings.

Although the non-replication of the three CpGs that survived FDR correction in NSHD could be related to cohort differences, the distribution of the p-values also suggests that there were no associations or we were underpowered to identify individual CpGs in each cohort (Supplementary Figure 5 and 6) even though smaller case-control studies previously reported evidence for associations^11,13,15^. Although selection bias—with the DNA methylation sample being slightly more affluent than the full cohort—may bias results towards the null^48^, in our study most of the ACEs had similar prevalence estimates in the DNA methylation subsample, suggesting selection bias is not likely to have affected our findings. One potential explanation for the contrast between findings in case-control studies and the lack of individual CpGs in this and other population-based cohort studies is that childhood adversity related DNA methylation differences are more subtle in population-based cohorts compared to high risk case-control designs due to differences in severity of the adversity exposures and the instruments used to assess childhood adversity. These differences may also explain the lack of enrichment of most previously identified CpGs in our population-based cohorts. In this context, it is striking that the most robust enrichment was for the Dunedin study, another population-based cohort study^18^. Here, one out of two CpGs associated with sexual abuse replicated in ALSPAC. Note that enrichment in NSHD could not be determined due to a lack of sexual abuse data. The Dunedin cohort had the most similarities to our ALSPAC sample: retrospectively reported sexual abuse exposure between 0–16 years in a relatively large (~800) population-based birth cohort sample, with DNA methylation assessed in mid-life.

Overall, we find evidence that adverse childhood experiences, particularly those related to parental health, can have long-term effects on peripheral DNA methylation in mid-life. We find some evidence to replicate a previously identified CpG site associated with sexual abuse.

### Code availability

Code to generate these results is available from the authors on request.

## Electronic supplementary material

Supplementary Material
