# Supplementary material for: Childhood adversity and DNA methylation in two population-based cohorts
Source: Transl Psychiatry. 2018 Dec 3;8:266. doi: 10.1038/s41398-018-0307-3 (PMC6277431; doi:10.1038/s41398-018-0307-3)
Supplement: Supplementary file 1 — Supplementary Material [file 41398_2018_307_MOESM1_ESM.doc]

**Supplementary material for:**

*Childhood adversity and adult DNA methylation in two population-based cohorts*

*L.C. Houtepen^1^, R Hardy^2^, J Maddock^2^, D. Kuh^2^, E.L. Anderson*^1^*, C.L. Relton^1^, M.J. Suderman*^1^, L.D. Howe*^1#^*

#Corresponding author: laura.howe@bristol.ac.uk. MRC Integrative Epidemiology Unit, University of Bristol, Oakfield House, Bristol BS8 2BN, UK. +44 (0)117 3310134
1 – MRC Integrative Epidemiology Unit at the University of Bristol, Population Health Sciences, Bristol Medical School, University of Bristol, Bristol, UK.
2 – MRC Unit for Lifelong Health and Ageing at University College London, London, UK.

*These authors contributed equally to this work

#Corresponding author: laura.howe@bristol.ac.uk. MRC Integrative Epidemiology Unit, University of Bristol, Oakfield House, Bristol BS8 2BN, UK. +44 (0)117 3310134

***Pre-processing of the DNA methylation data***

To account for technical variation, pre-processing was performed on the entire dataset (ALSPAC n=5469 including technical replicates and DNA samples from the children, see details (Min, Hemani, Davey Smith, Relton, & Suderman, 2017); NSHD n=996 including 810 buccal and 186 blood samples). Samples were removed if the reported sex did not match the methylation-predicted sex, the sample was an outlier on mean methylated and unmethylated channels, if >10% of the probes in the sample had a detection P-value>0.01 or >10% of the probes in the sample had a bead count less than three. In ALSPAC, 976 out of 1083 samples for the mothers at age 47 and in NSHD, 766 buccal and 153 blood samples survived quality control. Probes were removed if they failed quality control (detection P-value>0.01 for >10% of samples or bead count < 3 for >10% of samples) (ALSPAC n=3322, NSHD n=364), were nonspecific (Chen et al., 2013), were located on the Y chromosome or were one of the 65 single-nucleotide polymorphism (SNP) probes included on the array for quality control purposes, leaving 453 965 probes in ALSPAC and 455 971 probes in NSHD for further analysis.

*References*

Min J, Hemani G, Davey Smith G, Relton CL, Suderman M. Meffil: efficient normalisation and analysis of very large DNA methylation samples. *bioRxiv* 2017.http://biorxiv.org/content/early/2017/04/27/125963.abstract.

Chen YA, Lemire M, Choufani S, Butcher DT, Grafodatskaya D, Zanke BW *et al.* Discovery of cross-reactive probes and polymorphic CpGs in the Illumina Infinium HumanMethylation450 microarray. *Epigenetics* 2013; **8**: 203–209.

***Differentially methylated regions***

Using genomic location and P-values from the EWAS for the individual CpGs, Comb-P identifies regions that are enriched for low P-values. It then calculates and adjusts for auto-correlation between those P-values using the StoufferLiptak-Kechris correction and performs one-step Šidák correction for multiple-testing. Differentially methylated regions (DMRs) were defined as regions fulfilling these criteria: 1) contain at least two probes, 2) all probes within the region are within 500 base pairs of at least one other probe in the region, 3) the Šidák-corrected P-value for the region is <0.05.

***Childhood adversity definitions***

In both cohorts, eight different types of childhood adversity were considered: maltreatment [abuse or neglect of any kind], sub-optimal maternal bonding, parental physical illness, parental mental illness, parental divorce or separation and death of mother or father in childhood. In ALSPAC, more detailed information was available on type of maltreatment, this was used to split any maltreatment further into exposure to sexual abuse, physical abuse, emotional abuse, emotional neglect and physical neglect. Consequently, the adverse childhood experiences (ACE) score in NSHD is calculated based on seven different ACE types, while the ALSPAC ACE score is based on eleven different ACE types. In ALSPAC, women reported adverse childhood experiences retrospectively in questionnaires administered at the time of enrolment into the study (mean age 28 years), through pregnancy and postnatally (until their child was 3 years of age). In NSHD, adverse childhood experiences were reported in interviews and questionnaires prospectively at age 4 years (or at 7 or 11 years if missing), except for parental bonding and maltreatment which were recalled when participants were age 43. For both cohorts, the exact definitions used are presented in Supplementary Table 1.

***Supplementary Table 1*** *The adversity definitions applied in the ALSPAC and NSHD cohorts.*

| **Adversity** | ***ALSPAC*** | | ***NSHD*** | |
| --- | --- | --- | --- | --- |
|  | Items | Exposure definition | Items | Exposure definition |
| *Parent physically ill* | 1. Before age 17 was either parent seriously ill? 2. Was your mother disabled in any way? 3. Was your father disabled in any way? | Yes on any of the three items | In a health visitor interview mothers reported if they themselves or their partner had a serious physical or psychiatric illness during the first 15 years of their child's life | Any serious physical illness for either mother or father |
| *Child illness* | 1. Did you have a serious physical illness before age 17 years? | Yes | International Classification of Diseases (ICD) codes were recorded | Exposure to any of these disorders |
| *Parent mentally ill* | Was either parent mentally ill before age 17 years? | Yes | In a health visitor interview mothers reported if they themselves or their partner had a serious physical or psychiatric illness during the first 15 years of their child's life | Any serious psychiatric illness for either mother or father |
| *Sub optimal maternal bonding* | Adapted Parental Bonding Instrument (PBI) (Parker, Tupling, & Brown, 1979)^1^ consisting of 22 items with either three or two response categories per item  12 items on maternal care  10 items on maternal protection | Neglectful parenting (low care and high protection) based on the bottom 10^th^ percentile for care and the top 10^th^ percentile for protection | Adapted Parental Bonding Instrument (PBI) (Parker et al., 1979)^1^ consisting of 24 items with four response categories per item  11 items on maternal care  13 items on maternal protection | Neglectful parenting (low care and high protection) based on the bottom 10^th^ percentile for care and the top 10^th^ percentile for protection |
| *Parents separated* | 1. Before age 17 years were your parents separated or divorced? 2. Before age 17 years were your parents separated?   Before age 17 years were your parents divorced? | Yes on any of the three items | Experience of parental divorce and age at which it occurred was recorded | Parents divorced before participant was 17 years of age |
| *Parent died* | 1. Before age 17 years did your father die? 2. Before age 17 years did your mother die? 3. Before age 17 years did your father figure die? 4. Before age 17 years did your mother figure die? 5. Before age 17 years did your parent die? | Yes on any of the five items | Experience of parental death and age at which it occurred was recorded | Parent died before participant was 17 years of age |
| *Child maltreatment* | 1. Same five items as used for sexual abuse, physical abuse, emotional abuse, emotional neglect and physical neglect | Yes on any of the five items | As a child do you feel you were mistreated by your parents in any way? | Yes |
| *Sexual abuse* | Before age 17 you were sexually abused? | Yes | Not available |  |
| *Physical abuse* | Before age 17 was either parent physically cruel to you? | Yes | Not available | |
| *Emotional abuse* | Before age 17 was either parent emotionally cruel to you? | Yes | Not available | |
| *Physical neglect* | Were you physically neglected as a child? | Yes | Not available | |
| *Emotional neglect* | Were you emotionally neglected as a child? | Yes | Not available | |
|  |  |  |  | |

^1^Parker G, Tupling H, Brown LB. A Parental Bonding Instrument. *Br J Med Psychol* 1979; **52**: 1–10.

***Prevalence of adversity comparing included participants versus the full cohorts, and comparing ALSPAC and NSHD***

In ALSPAC, 8021 out of 14541 participants had valid data for the eleven adversity measures during childhood. This study included the 780 ALSPAC participants that also had blood DNA methylation measured, which is a subcohort with a similar distribution of the ACE score and similar prevalence for child illness, sexual, physical and emotional abuse as well as physical and emotional neglect, but contains less current smokers and has a lower prevalence for separation parents, death of parent and suboptimal maternal bond as well as a slightly higher prevalence of mental illness than the entire ALSPAC cohort (see Supplementary Table 2 for a comparison between the included and excluded sample).

In NSHD, 569 out of 2230 participants had valid data for the seven psychosocial adversity measures during childhood. For the main analyses, this study included the 552 participants that also had buccal DNA methylation measured. The buccal DNA methylation subcohort is similar to the entire NSHD cohort, except for lower smoking rates, higher parental physical illness, lower parental separation, lower parental death and lower childhood illness (see Supplementary Table 3 for a comparison between the included and excluded sample). Childhood adversity prevalence in the 98 NSHD participants with buccal as well as white blood cell (WBC) DNA methylation measured was comparable to the prevalence in the larger buccal DNA methylation cohort (n=552), although no one reported parental mental illness which could therefore not be examined in the tissue comparison.

Finally, the prevalence of the different adverse childhood experiences (ACEs) in the participants with complete adversity data in NSHD and ALSPAC as well as the methylation subsets of NSHD and ALSPAC is depicted in Supplementary Figure 1. When comparing the NSHD methylation cohort (n=552) with the ALSPAC methylation cohort (n=780), prevalence of parental death and physical illness are comparable, while parental mental illness, separation of the parents and any childhood maltreatment are less prevalent in NSHD than ALSPAC and a suboptimal maternal bond or child illness are more prevalent in NSHD then ALSPAC.


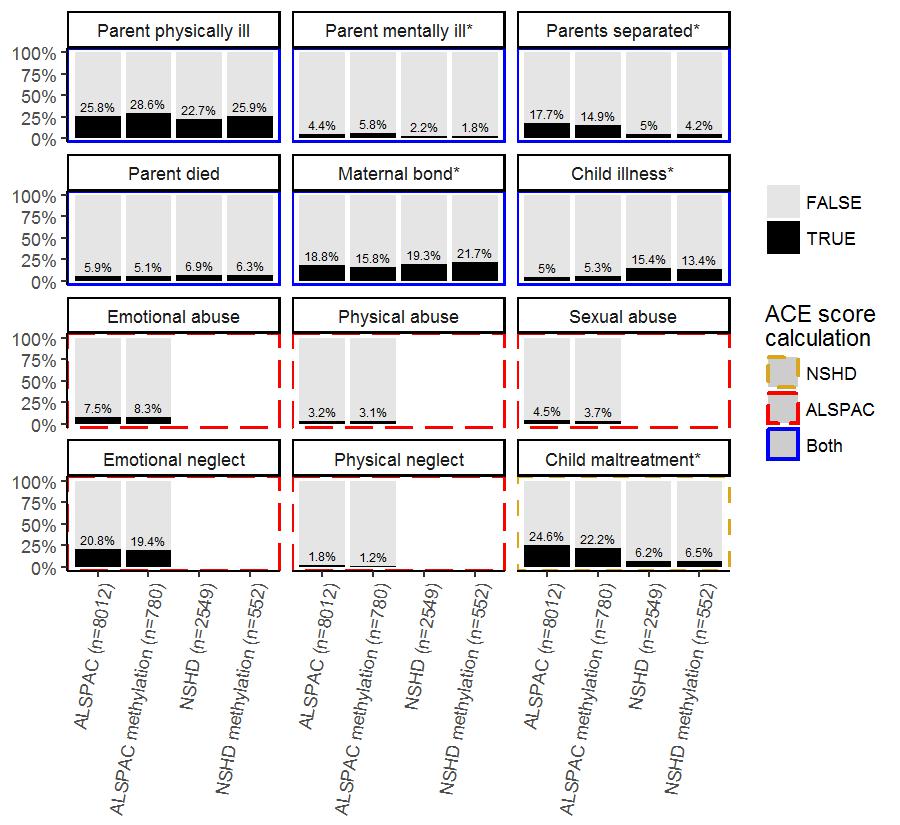


**Supplementary Figure 1** *The percentage of individuals exposed to a specific type of childhood adverse experience (ACE) in the ALSPAC cohort with complete adversity data, the methylation subset in ALSPAC, the NSHD cohort with complete adversity data or the methylation subcohort in NSHD. The colour and line type around each panel indicate whether the ACE subtype was used in the ACE score calculation in both ALSPAC and NSHD, only in NSHD or only in ALSPAC. Finally, the * in plot titles indicates whether the ALSPAC and NSHD cohorts had a significantly different prevalence (Chi-square test P < 0.05).*

*
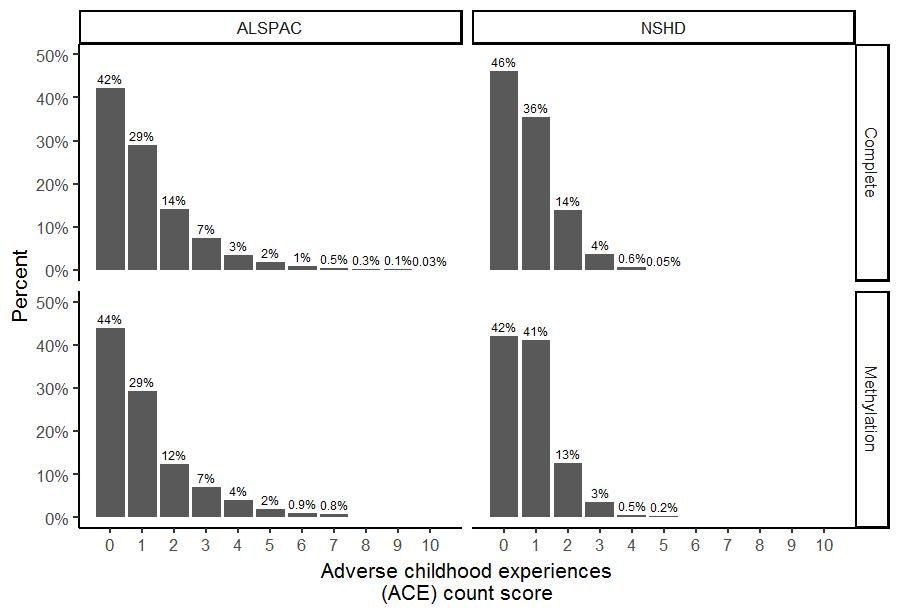
*

**Supplementary Figure 2** *The distribution for the childhood adverse experience (ACE) count score in the ALSPAC cohort with complete adversity data (n=8021), the methylation subcohort in ALSPAC (n=780), the NSHD cohort with complete adversity data (n=2438) or the methylation subcohort in NSHD (n=552).*

***Supplementary Table 2*** *Comparison of included (n=780) and excluded participants in the ALSPAC data. Excluded participants are those that had missing data for either one of the adversity exposure variables or DNA methylation. Adversity measures in shaded boxes are the binary constructs used in EWAS analyses in this manuscript; the measures in unshaded boxes below each adversity construct are the questionnaire items used to construct the binary constructs.*

| **Description (n with available data)** | **Excluded** | **Included** | **p** (pearson chi squared) |
| --- | --- | --- | --- |
| Total sample size(n=14541) | 13761 | 780 |  |
| Sex(n=14541) = Female (%) | 13761 (100.0) | 780 (100.0) | NA |
| Age clinic(n=4735) (mean (sd)) | 47.87 (4.53) | 47.93 (4.19) | 0.749 |
| Smoking(n=3996) (%) |  |  | 0.006 |
| Never-smoker | 1818 (52.6) | 303 (56.3) |  |
| Ex-smoker | 1222 (35.3) | 195 (36.2) |  |
| Current-smoker | 418 (12.1) | 40 (7.4) |  |
| Currently a smoker(n=4123) (%) | 418 (11.7) | 40 (7.2) | 0.002 |
| Respondent ever smoked in the past(n=3553) (%) | 1234 (40.4) | 195 (39.2) | 0.637 |
| **Parent physically ill(n=11639)(%)** | 2939 (27.1) | 223 (28.6) | 0.377 |
| Disabled mother (n=12318) (%) | 692 (6.0) | 54 (6.9) | 0.322 |
| Disabled father (n=12151) (%) | 840 (7.4) | 71 (9.1) | 0.089 |
| Before 17 parent seriously ill (n=12286) (%) |  |  | 0.677 |
| No | 9468 (82.3) | 633 (81.2) |  |
| Yes but no effect | 188 (1.6) | 10 (1.3) |  |
| Yes mild effect | 477 (4.1) | 38 (4.9) |  |
| Yes moderate effect | 582 (5.1) | 45 (5.8) |  |
| Yes big effect | 791 (6.9) | 54 (6.9) |  |
| **Parent mentally ill(n=12286)(%)** | 471 (4.1) | 45 (5.8) | 0.03 |
| Before 17 parents mentally ill (n=12286) (%) |  |  | 0.008 |
| No | 11035 (95.9) | 735 (94.2) |  |
| Yes but no effect | 62 (0.5) | 1 (0.1) |  |
| Yes mild effect | 129 (1.1) | 12 (1.5) |  |
| Yes moderate effect | 127 (1.1) | 18 (2.3) |  |
| Yes big effect | 153 (1.3) | 14 (1.8) |  |
| **Parents separated(n=11863)(%)** | 2578 (23.3) | 116 (14.9) | <0.001 |
| Before 17 parents separated (n=12286) (%) |  |  | 0.108 |
| No | 9517 (82.7) | 673 (86.3) |  |
| Yes but no effect | 287 (2.5) | 13 (1.7) |  |
| Yes mild effect | 374 (3.3) | 24 (3.1) |  |
| Yes moderate effect | 539 (4.7) | 30 (3.8) |  |
| Yes big effect | 789 (6.9) | 40 (5.1) |  |
| Before 17 parents divorced (n=12286) (%) |  |  | 0.028 |
| No | 9702 (84.3) | 690 (88.5) |  |
| Yes but no effect | 329 (2.9) | 13 (1.7) |  |
| Yes mild effect | 360 (3.1) | 17 (2.2) |  |
| Yes moderate effect | 467 (4.1) | 28 (3.6) |  |
| Yes big effect | 648 (5.6) | 32 (4.1) |  |
| Parents divorced or separated before your 18th birthday (n=12231) (%) | 2265 (19.8) | 106 (13.6) | <0.001 |
| **Parent died(n=11742)(%)** | 794 (7.2) | 40 (5.1) | 0.032 |
| <17 when mother died (n=12444) (%) | 208 (1.8) | 10 (1.3) | 0.372 |
| <17 when father died (n=12444) (%) | 464 (4.0) | 26 (3.3) | 0.423 |
| <17 when mother figure died (n=12444) (%) | 32 (0.3) | 1 (0.1) | 0.683 |
| <17 when father figure died (n=12444) (%) | 35 (0.3) | 2 (0.3) | 1 |
| Before 17 parent died (n=12286) (%) |  |  | 0.357 |
| No | 10815 (94.0) | 743 (95.3) |  |
| Yes but no effect | 45 (0.4) | 0 (0.0) |  |
| Yes mild effect | 61 (0.5) | 4 (0.5) |  |
| Yes moderate effect | 105 (0.9) | 5 (0.6) |  |
| Yes big effect | 480 (4.2) | 28 (3.6) |  |
| **Sub optimal maternal bonding(n=11124)(%)** | 2047 (19.8) | 123 (15.8) | 0.007 |
| Parental Bonding Instrument(PBI)- Maternal care score(n=11427) (mean (sd)) | 19.76 (5.50) | 20.12 (5.23) | 0.076 |
| Spoken to warmly by mother(n=12169) (%) |  |  | 0.009 |
| Usually | 8951 (78.6) | 649 (83.2) |  |
| Sometimes | 2246 (19.7) | 121 (15.5) |  |
| Never | 192 (1.7) | 10 (1.3) |  |
| Helped as needed by mother (n=12206) (%) |  |  | 0.039 |
| Usually | 9143 (80.0) | 652 (83.6) |  |
| Sometimes | 1972 (17.3) | 114 (14.6) |  |
| Never | 311 (2.7) | 14 (1.8) |  |
| Apparent coldness from mother (n=12181) (%) |  |  | 0.891 |
| Never | 8692 (76.2) | 599 (76.8) |  |
| Sometimes | 2153 (18.9) | 142 (18.2) |  |
| Usually | 556 (4.9) | 39 (5.0) |  |
| Problems seemed understood by mother (n=12214) (%) |  |  | 0.313 |
| Don`t know | 1 (0.0) | 0 (0.0) |  |
| Usually | 5748 (50.3) | 415 (53.2) |  |
| Sometimes | 4760 (41.6) | 313 (40.1) |  |
| Never | 925 (8.1) | 52 (6.7) |  |
| Affection by mother (n=12208) (%) |  |  | 0.178 |
| Usually | 7986 (69.9) | 569 (72.9) |  |
| Sometimes | 2914 (25.5) | 181 (23.2) |  |
| Never | 528 (4.6) | 30 (3.8) |  |
| Felt unwanted by mother (n=12215) (%) |  |  | 0.048 |
| Never | 9523 (83.3) | 673 (86.3) |  |
| Sometimes | 1454 (12.7) | 87 (11.2) |  |
| Usually | 458 (4.0) | 20 (2.6) |  |
| Things talked over by mother (n=12219) (%) |  |  | 0.244 |
| Usually | 5067 (44.3) | 354 (45.4) |  |
| Sometimes | 5221 (45.6) | 362 (46.4) |  |
| Never | 1151 (10.1) | 64 (8.2) |  |
| Praised by mother (n=12188) (%) |  |  | 0.001 |
| Usually | 5891 (51.6) | 455 (58.4) |  |
| Sometimes | 4728 (41.4) | 282 (36.2) |  |
| Never | 790 (6.9) | 42 (5.4) |  |
| Mother enjoyed talking things over (n=11994) = No (%) | 2210 (19.7) | 139 (17.8) | 0.223 |
| Frequently smiled at by mother (n=12100) = No (%) | 1194 (10.5) | 81 (10.4) | 0.944 |
| Needs understood by mother (n=11977) = No (%) | 2510 (22.4) | 147 (18.9) | 0.024 |
| Upsets comforted by mother (n=12040) = No (%) | 1569 (13.9) | 84 (10.8) | 0.016 |
| Parental Bonding Instrument (PBI)- Maternal overprotection score (n=11541) (mean (sd)) | 6.47 (4.17) | 6.16 (4.05) | 0.048 |
| Allowed by mother to do as liked(n=12232) (%) |  |  | 0.013 |
| Usually | 6611 (57.7) | 465 (59.6) |  |
| Sometimes | 4617 (40.3) | 311 (39.9) |  |
| Never | 224 (2.0) | 4 (0.5) |  |
| Control attempted by mother (n=12201) (%) |  |  | 0.868 |
| Never | 1513 (13.2) | 101 (12.9) |  |
| Sometimes | 7016 (61.4) | 475 (60.9) |  |
| Usually | 2892 (25.3) | 204 (26.2) |  |
| Privacy invaded by mother (n=12185) (%) |  |  | 0.023 |
| Never | 5978 (52.4) | 439 (56.3) |  |
| Sometimes | 4483 (39.3) | 295 (37.8) |  |
| Usually | 944 (8.3) | 46 (5.9) |  |
| Allowed own decisions by mother d708(n=12213) (%) |  |  | <0.001 |
| Usually | 5134 (44.9) | 396 (50.8) |  |
| Sometimes | 5754 (50.3) | 367 (47.1) |  |
| Never | 545 (4.8) | 17 (2.2) |  |
| Allowed freedom by mother d711(n=12209) (%) |  |  | 0.003 |
| Don`t know | 1 (0.0) | 0 (0.0) |  |
| Usually | 4065 (35.6) | 306 (39.2) |  |
| Sometimes | 6497 (56.8) | 441 (56.5) |  |
| Never | 866 (7.6) | 33 (4.2) |  |
| Babied by mother (n=12137) (%) | 2245 (19.8) | 114 (14.6) | 0.001 |
| Felt helpless without mother (n=12093) (%) | 1792 (15.8) | 97 (12.4) | 0.013 |
| Allowed by mother to go out anytime (n=12000) (%) | 5138 (45.8) | 343 (44.0) | 0.36 |
| Overprotective mother (n=12058) (%) | 2378 (21.1) | 158 (20.3) | 0.614 |
| Allowed by mother to dress as liked (n=12037) = No (%) | 4754 (42.2) | 351 (45.0) | 0.14 |
| **Child illness(n=12286)(%)** | 549 (4.8) | 41 (5.3) | 0.599 |
| Had serious physical illness before 17 (n=12286) (%) |  |  | 0.967 |
| No | 10957 (95.2) | 739 (94.7) |  |
| Yes but no effect | 112 (1.0) | 8 (1.0) |  |
| Yes mild effect | 132 (1.1) | 11 (1.4) |  |
| Yes moderate effect | 142 (1.2) | 10 (1.3) |  |
| Yes big effect | 163 (1.4) | 12 (1.5) |  |
| Child maltreatment(n=9726)(%) | 2660 (29.7) | 173 (22.2) | <0.001 |
| **Sexual abuse(n=12286)(%)** | 599 (5.2) | 29 (3.7) | 0.081 |
| Sexually abused before 17(n=12286) (%) |  |  | 0.263 |
| No | 10907 (94.8) | 751 (96.3) |  |
| Yes but no effect | 36 (0.3) | 0 (0.0) |  |
| Yes mild effect | 88 (0.8) | 5 (0.6) |  |
| Yes moderate effect | 123 (1.1) | 8 (1.0) |  |
| Yes big effect | 352 (3.1) | 16 (2.1) |  |
| **Physical abuse(n=12286)(%)** | 397 (3.5) | 24 (3.1) | 0.65 |
| Parent physically cruel before 17(n=12286) (%) |  |  | 0.395 |
| No | 11109 (96.5) | 756 (96.9) |  |
| Yes but no effect | 33 (0.3) | 1 (0.1) |  |
| Yes mild effect | 76 (0.7) | 8 (1.0) |  |
| Yes moderate effect | 112 (1.0) | 8 (1.0) |  |
| Yes big effect | 176 (1.5) | 7 (0.9) |  |
| **Emotional abuse(n=12286)(%)** | 873 (7.6) | 65 (8.3) | 0.49 |
| Parent emotionally cruel before 17(n=12286) (%) |  |  | 0.415 |
| No | 10633 (92.4) | 715 (91.7) |  |
| Yes but no effect | 52 (0.5) | 1 (0.1) |  |
| Yes mild effect | 214 (1.9) | 17 (2.2) |  |
| Yes moderate effect | 248 (2.2) | 16 (2.1) |  |
| Yes big effect | 359 (3.1) | 31 (4.0) |  |
| **Physical neglect(n=9590)(%)** | 181 (2.1) | 9 (1.2) | 0.11 |
| Mum physically neglected when child(n=9590) (%) |  |  | 0.145 |
| no not at all | 8629 (97.9) | 771 (98.8) |  |
| yes somewhat | 156 (1.8) | 9 (1.2) |  |
| yes severely | 25 (0.3) | 0 (0.0) |  |
| **Emotional neglect(n=9579)(%)** | 1945 (22.1) | 151 (19.4) | 0.083 |
| Mum emotionally neglected when child(n=9579) (%) |  |  | 0.017 |
| no not at all | 6854 (77.9) | 629 (80.6) |  |
| yes somewhat | 1703 (19.4) | 142 (18.2) |  |
| yes severely | 242 (2.8) | 9 (1.2) |  |
| **Adverse Childhood Experiences (ACE) count score(n=8021) (%)** |  |  | 0.676 |
| 0 | 3057 (42.2) | 342 (43.8) |  |
| 1 | 2094 (28.9) | 229 (29.4) |  |
| 2 | 1023 (14.1) | 96 (12.3) |  |
| 3 | 542 (7.5) | 54 (6.9) |  |
| 4 | 252 (3.5) | 31 (4.0) |  |
| 5 | 137 (1.9) | 15 (1.9) |  |
| 6 | 72 (1.0) | 7 (0.9) |  |
| 7 | 34 (0.5) | 6 (0.8) |  |
| 8 | 19 (0.3) | 0 (0.0) |  |
| 9 | 9 (0.1) | 0 (0.0) |  |
| 10 | 2 (0.0) | 0 (0.0) |  |

**Supplementary Table 3** *Comparison of included (n=552) and excluded participants in the NSHD data. Excluded participants are those that had missing data for either one of the adversity exposure variables or no buccal DNA methylation measurement. Adversity measures in shaded boxes are the binary constructs used in EWAS analyses in this manuscript; the measures in unshaded boxes below each adversity construct are the questionnaire items used to construct the binary constructs.*

| **Description (n with available data)** | **Excluded** | **Included** | **p** (pearson chi squared) |
| --- | --- | --- | --- |
| Total sample size (n=5362) | 4810 | 552 |  |
| Sex(n=5362) = Female (%) | 1995 (41.5) | 552 (100.0) | <0.001 |
| Age clinic(n=2988) (mean (sd)) | 53.50 (0.50) | 53.45 (0.50) | 0.074 |
| Smoking - history up to 53 years(n=2988) (%) |  |  | 0.017 |
| Never-smoker | 691 (28.4) | 184 (33.3) |  |
| Ex-smoker | 1157 (47.5) | 261 (47.3) |  |
| Current-smoker | 588 (24.1) | 107 (19.4) |  |
| **Parent physically ill(n=5362) (%)** | 970 (20.2) | 143 (25.9) | 0.002 |
| **Parent mentally ill(n=5362) (%)** | 103 (2.1) | 10 (1.8) | 0.723 |
| Type of illness of father up until the child was 15 years old (n=5362) (%) |  |  | 0.087 |
| Father not ill | 4077 (84.8) | 443 (80.3) |  |
| Unknown cause | 8 (0.2) | 0 (0.0) |  |
| Psychiatric | 50 (1.0) | 7 (1.3) |  |
| Headache, migraine | 10 (0.2) | 1 (0.2) |  |
| Arthritis | 25 (0.5) | 1 (0.2) |  |
| Skin | 8 (0.2) | 2 (0.4) |  |
| Peptic ulcer | 118 (2.5) | 14 (2.5) |  |
| Other now malignant alimentary | 53 (1.1) | 10 (1.8) |  |
| Endocrine | 20 (0.4) | 3 (0.5) |  |
| Degenerative C.V.S. | 38 (0.8) | 2 (0.4) |  |
| Respiratory | 107 (2.2) | 17 (3.1) |  |
| C.N.S. | 27 (0.6) | 6 (1.1) |  |
| Defective vision and/or hearing | 13 (0.3) | 6 (1.1) |  |
| Malignant | 13 (0.3) | 1 (0.2) |  |
| Accident | 78 (1.6) | 14 (2.5) |  |
| Physical handicap, deformity | 18 (0.4) | 4 (0.7) |  |
| Other | 124 (2.6) | 18 (3.3) |  |
| Indeterminate | 11 (0.2) | 2 (0.4) |  |
| Heart (not degenerative) | 12 (0.2) | 1 (0.2) |  |
| Type of illness of mother up until the child was 15 years old (n=5362) (%) |  |  | 0.887 |
| Mother not ill | 4336 (90.1) | 494 (89.5) |  |
| Unknown cause | 1 (0.0) | 0 (0.0) |  |
| Psychiatric | 56 (1.2) | 3 (0.5) |  |
| Headache, migraine | 22 (0.5) | 4 (0.7) |  |
| Arthritis | 12 (0.2) | 1 (0.2) |  |
| Skin | 10 (0.2) | 2 (0.4) |  |
| Obstetric or Gynaecological | 101 (2.1) | 16 (2.9) |  |
| Peptic ulcer | 19 (0.4) | 2 (0.4) |  |
| Other non-malignant ailment | 27 (0.6) | 3 (0.5) |  |
| Endocrine | 38 (0.8) | 3 (0.5) |  |
| Degenerative C.V.S | 13 (0.3) | 3 (0.5) |  |
| Respiratory | 40 (0.8) | 8 (1.4) |  |
| C.N.S | 16 (0.3) | 1 (0.2) |  |
| Defective vision and/or hearing | 10 (0.2) | 0 (0.0) |  |
| Malignant | 12 (0.2) | 2 (0.4) |  |
| Accident | 11 (0.2) | 0 (0.0) |  |
| Physical Handicap | 6 (0.1) | 1 (0.2) |  |
| Other | 65 (1.4) | 8 (1.4) |  |
| Indeterminate | 2 (0.0) | 0 (0.0) |  |
| Heart (not degenerative) | 13 (0.3) | 1 (0.2) |  |
| **Parents separated(n=5360) (%)** | 292 (6.1) | 23 (4.2) | 0.088 |
| Age in years at which parents divorced(n=5360) (%) |  |  | 0.836 |
| n/a | 4461 (92.8) | 523 (94.7) |  |
| 0 | 26 (0.5) | 1 (0.2) |  |
| 1 | 25 (0.5) | 2 (0.4) |  |
| 2 | 35 (0.7) | 3 (0.5) |  |
| 3 | 25 (0.5) | 2 (0.4) |  |
| 4 | 31 (0.6) | 1 (0.2) |  |
| 5 | 17 (0.4) | 0 (0.0) |  |
| 6 | 15 (0.3) | 1 (0.2) |  |
| 7 | 18 (0.4) | 0 (0.0) |  |
| 8 | 17 (0.4) | 3 (0.5) |  |
| 9 | 11 (0.2) | 2 (0.4) |  |
| 10 | 10 (0.2) | 3 (0.5) |  |
| 11 | 5 (0.1) | 0 (0.0) |  |
| 12 | 12 (0.2) | 1 (0.2) |  |
| 13 | 15 (0.3) | 0 (0.0) |  |
| 14 | 8 (0.2) | 1 (0.2) |  |
| 15 | 11 (0.2) | 1 (0.2) |  |
| 16 | 7 (0.1) | 2 (0.4) |  |
| 17 | 4 (0.1) | 0 (0.0) |  |
| 18 | 7 (0.1) | 0 (0.0) |  |
| 19 | 7 (0.1) | 1 (0.2) |  |
| 20 | 8 (0.2) | 1 (0.2) |  |
| 21 | 8 (0.2) | 1 (0.2) |  |
| 22 | 7 (0.1) | 0 (0.0) |  |
| 23 | 7 (0.1) | 1 (0.2) |  |
| 24 | 5 (0.1) | 1 (0.2) |  |
| 25 | 2 (0.0) | 1 (0.2) |  |
| 26 | 4 (0.1) | 0 (0.0) |  |
| **Parent died(n=4943) (%)** | 370 (8.4) | 35 (6.3) | 0.109 |
| Age in years at death of father (n=5159) (%) |  |  | 0.006 |
| n/a | 4004 (86.8) | 515 (94.7) |  |
| 0 | 13 (0.3) | 0 (0.0) |  |
| 1 | 10 (0.2) | 1 (0.2) |  |
| 2 | 6 (0.1) | 1 (0.2) |  |
| 3 | 9 (0.2) | 1 (0.2) |  |
| 4 | 14 (0.3) | 1 (0.2) |  |
| 5 | 10 (0.2) | 0 (0.0) |  |
| 6 | 11 (0.2) | 2 (0.4) |  |
| 7 | 17 (0.4) | 1 (0.2) |  |
| 8 | 14 (0.3) | 1 (0.2) |  |
| 9 | 18 (0.4) | 1 (0.2) |  |
| 10 | 19 (0.4) | 0 (0.0) |  |
| 11 | 15 (0.3) | 2 (0.4) |  |
| 12 | 8 (0.2) | 2 (0.4) |  |
| 13 | 18 (0.4) | 4 (0.7) |  |
| 14 | 18 (0.4) | 2 (0.4) |  |
| 15 | 18 (0.4) | 3 (0.6) |  |
| 16 | 19 (0.4) | 2 (0.4) |  |
| 17 | 24 (0.5) | 2 (0.4) |  |
| 18 | 32 (0.7) | 0 (0.0) |  |
| 19 | 34 (0.7) | 0 (0.0) |  |
| 20 | 35 (0.8) | 2 (0.4) |  |
| 21 | 44 (1.0) | 0 (0.0) |  |
| 22 | 33 (0.7) | 0 (0.0) |  |
| 23 | 53 (1.1) | 0 (0.0) |  |
| 24 | 53 (1.1) | 0 (0.0) |  |
| 25 | 50 (1.1) | 0 (0.0) |  |
| 26 | 14 (0.3) | 1 (0.2) |  |
| 27 | 2 (0.0) | 0 (0.0) |  |
| Age in years at death of mother (n=4777) (%) |  |  | 0.598 |
| n/a | 4004 (94.2) | 515 (97.7) |  |
| 0 | 3 (0.1) | 0 (0.0) |  |
| 1 | 7 (0.2) | 1 (0.2) |  |
| 2 | 4 (0.1) | 0 (0.0) |  |
| 3 | 7 (0.2) | 1 (0.2) |  |
| 4 | 8 (0.2) | 0 (0.0) |  |
| 5 | 6 (0.1) | 1 (0.2) |  |
| 6 | 6 (0.1) | 0 (0.0) |  |
| 7 | 6 (0.1) | 0 (0.0) |  |
| 8 | 2 (0.0) | 0 (0.0) |  |
| 9 | 6 (0.1) | 0 (0.0) |  |
| 10 | 6 (0.1) | 1 (0.2) |  |
| 11 | 9 (0.2) | 0 (0.0) |  |
| 12 | 9 (0.2) | 0 (0.0) |  |
| 13 | 4 (0.1) | 2 (0.4) |  |
| 14 | 14 (0.3) | 3 (0.6) |  |
| 15 | 6 (0.1) | 1 (0.2) |  |
| 16 | 9 (0.2) | 0 (0.0) |  |
| 17 | 6 (0.1) | 0 (0.0) |  |
| 18 | 9 (0.2) | 0 (0.0) |  |
| 19 | 10 (0.2) | 0 (0.0) |  |
| 20 | 8 (0.2) | 0 (0.0) |  |
| 21 | 11 (0.3) | 0 (0.0) |  |
| 22 | 12 (0.3) | 0 (0.0) |  |
| 23 | 24 (0.6) | 0 (0.0) |  |
| 24 | 24 (0.6) | 1 (0.2) |  |
| 25 | 24 (0.6) | 1 (0.2) |  |
| 26 | 5 (0.1) | 0 (0.0) |  |
| 27 | 1 (0.0) | 0 (0.0) |  |
| **Sub optimal maternal bonding(n=2965) (%)** | 452 (18.7) | 120 (21.7) | 0.12 |
| Parental Bonding Instrument (PBI)- Maternal care score (n=3066) (mean (sd)) | 24.85 (6.63) | 24.71 (7.04) | 0.672 |
| My mother spoke to me with a warm and friendly voice (n=3163) (%) |  |  | 0.864 |
| Moderately like this | 942 (36.1) | 194 (35.3) |  |
| Moderately unlike this | 107 (4.1) | 20 (3.6) |  |
| Very like this | 1513 (57.9) | 327 (59.5) |  |
| Very unlike this | 51 (2.0) | 9 (1.6) |  |
| My mother helped me as much as I needed (n=3119) (%) |  |  | 0.632 |
| Moderately like this | 774 (30.0) | 155 (28.5) |  |
| Moderately unlike this | 152 (5.9) | 35 (6.4) |  |
| Very like this | 1595 (61.9) | 345 (63.5) |  |
| Very unlike this | 55 (2.1) | 8 (1.5) |  |
| My mother appeared to understand my problems and worries (n=3122) (%) |  |  | 0.145 |
| Moderately like this | 1023 (39.7) | 196 (36.0) |  |
| Moderately unlike this | 349 (13.5) | 92 (16.9) |  |
| Very like this | 1081 (41.9) | 228 (41.8) |  |
| Very unlike this | 124 (4.8) | 29 (5.3) |  |
| My mother was affectionate to me (n=3106) (%) |  |  | 0.078 |
| Moderately like this | 798 (31.1) | 166 (30.6) |  |
| Moderately unlike this | 221 (8.6) | 57 (10.5) |  |
| Very like this | 1452 (56.6) | 289 (53.3) |  |
| Very unlike this | 93 (3.6) | 30 (5.5) |  |
| My mother enjoyed talking things over with me (n=3109) (%) |  |  | 0.574 |
| Moderately like this | 955 (37.3) | 195 (35.6) |  |
| Moderately unlike this | 546 (21.3) | 108 (19.7) |  |
| Very like this | 820 (32.0) | 187 (34.2) |  |
| Very unlike this | 241 (9.4) | 57 (10.4) |  |
| My mother frequently smiled at me (n=3098) (%) |  |  | 0.957 |
| Moderately like this | 788 (30.9) | 175 (31.9) |  |
| Moderately unlike this | 267 (10.5) | 58 (10.6) |  |
| Very like this | 1351 (53.0) | 286 (52.2) |  |
| Very unlike this | 144 (5.6) | 29 (5.3) |  |
| My mother seemed to understand what I needed or wanted (n=3098) (%) |  |  | 0.586 |
| Moderately like this | 1182 (46.3) | 248 (45.4) |  |
| Moderately unlike this | 498 (19.5) | 113 (20.7) |  |
| Very like this | 713 (27.9) | 144 (26.4) |  |
| Very unlike this | 159 (6.2) | 41 (7.5) |  |
| My mother made me feel I wasn't wanted (n=3081) (%) |  |  | 0.529 |
| Moderately like this | 208 (8.2) | 51 (9.4) |  |
| Moderately unlike this | 293 (11.6) | 72 (13.2) |  |
| Very like this | 168 (6.6) | 34 (6.2) |  |
| Very unlike this | 1867 (73.6) | 388 (71.2) |  |
| My mother could make me feel better when I was upset (n=3116) (%) |  |  | 0.584 |
| Moderately like this | 1003 (39.1) | 197 (35.9) |  |
| Moderately unlike this | 277 (10.8) | 60 (10.9) |  |
| Very like this | 1160 (45.2) | 263 (48.0) |  |
| Very unlike this | 128 (5.0) | 28 (5.1) |  |
| My mother talked to me often (n=3102) (%) |  |  | 0.063 |
| Moderately like this | 877 (34.3) | 173 (31.9) |  |
| Moderately unlike this | 307 (12.0) | 82 (15.1) |  |
| Very like this | 1211 (47.3) | 264 (48.6) |  |
| Very unlike this | 164 (6.4) | 24 (4.4) |  |
| My mother praised me (n=3123) (%) |  |  | 0.309 |
| Moderately like this | 1233 (47.9) | 241 (43.9) |  |
| Moderately unlike this | 362 (14.1) | 80 (14.6) |  |
| Very like this | 822 (31.9) | 187 (34.1) |  |
| Very unlike this | 157 (6.1) | 41 (7.5) |  |
| Parental Bonding Instrument (PBI)- Maternal overprotection score (n=2994) (mean (sd)) | 12.83 (6.63) | 13.38 (6.70) | 0.08 |
| My mother let me decide things for myself (n=3101) (%) |  |  | 0.377 |
| Moderately like this | 1180 (46.2) | 268 (49.0) |  |
| Moderately unlike this | 361 (14.1) | 83 (15.2) |  |
| Very like this | 824 (32.3) | 156 (28.5) |  |
| Very unlike this | 189 (7.4) | 40 (7.3) |  |
| My mother liked me to make my own decisions (n=3099) (%) |  |  | 0.849 |
| Moderately like this | 1171 (45.9) | 253 (46.3) |  |
| Moderately unlike this | 353 (13.8) | 82 (15.0) |  |
| Very like this | 920 (36.0) | 188 (34.4) |  |
| Very unlike this | 109 (4.3) | 23 (4.2) |  |
| My mother wanted me to grow up (n=3049) (%) |  |  | 0.305 |
| Moderately like this | 1164 (46.4) | 258 (47.5) |  |
| Moderately unlike this | 473 (18.9) | 117 (21.5) |  |
| Very like this | 709 (28.3) | 136 (25.0) |  |
| Very unlike this | 160 (6.4) | 32 (5.9) |  |
| My mother tried to control everything I did (n=3101) (%) |  |  | 0.29 |
| Moderately like this | 641 (25.1) | 155 (28.2) |  |
| Moderately unlike this | 881 (34.5) | 172 (31.3) |  |
| Very like this | 211 (8.3) | 51 (9.3) |  |
| Very unlike this | 819 (32.1) | 171 (31.1) |  |
| My mother invaded my privacy (n=3075) (%) |  |  | 0.553 |
| Moderately like this | 416 (16.4) | 100 (18.3) |  |
| Moderately unlike this | 791 (31.3) | 158 (28.9) |  |
| Very like this | 202 (8.0) | 48 (8.8) |  |
| Very unlike this | 1120 (44.3) | 240 (44.0) |  |
| My mother tended to baby me (n=3079) (%) |  |  | 0.024 |
| Moderately like this | 495 (19.5) | 84 (15.5) |  |
| Moderately unlike this | 733 (28.9) | 166 (30.6) |  |
| Very like this | 247 (9.7) | 40 (7.4) |  |
| Very unlike this | 1061 (41.8) | 253 (46.6) |  |
| My mother let me do those things I liked doing (n=3125) (%) |  |  | 0.888 |
| Moderately like this | 1269 (49.2) | 274 (50.3) |  |
| Moderately unlike this | 218 (8.4) | 48 (8.8) |  |
| Very like this | 1030 (39.9) | 212 (38.9) |  |
| Very unlike this | 63 (2.4) | 11 (2.0) |  |
| My mother tried to make me dependent on her (n=3108) (%) |  |  | 0.738 |
| Moderately like this | 404 (15.8) | 89 (16.2) |  |
| Moderately unlike this | 649 (25.4) | 150 (27.3) |  |
| Very like this | 204 (8.0) | 45 (8.2) |  |
| Very unlike this | 1301 (50.9) | 266 (48.4) |  |
| My mother felt I could not look after myself unless she was around (n=3085) (%) |  |  | 0.666 |
| Moderately like this | 398 (15.7) | 79 (14.5) |  |
| Moderately unlike this | 627 (24.7) | 141 (25.9) |  |
| Very like this | 183 (7.2) | 33 (6.1) |  |
| Very unlike this | 1333 (52.5) | 291 (53.5) |  |
| My mother gave me as much freedom as I wanted (n=3124) (%) |  |  | <0.001 |
| Moderately like this | 1145 (44.4) | 266 (48.5) |  |
| Moderately unlike this | 615 (23.9) | 152 (27.7) |  |
| Very like this | 568 (22.0) | 64 (11.7) |  |
| Very unlike this | 248 (9.6) | 66 (12.0) |  |
| My mother let me go out as often as I wanted (n=3114) (%) |  |  | <0.001 |
| Moderately like this | 1157 (45.1) | 244 (44.4) |  |
| Moderately unlike this | 586 (22.8) | 151 (27.5) |  |
| Very like this | 525 (20.5) | 63 (11.5) |  |
| Very unlike this | 297 (11.6) | 91 (16.6) |  |
| My mother was overprotective of me (n=3109) (%) |  |  | 0.702 |
| Moderately like this | 570 (22.3) | 115 (20.9) |  |
| Moderately unlike this | 764 (29.8) | 161 (29.3) |  |
| Very like this | 225 (8.8) | 56 (10.2) |  |
| Very unlike this | 1001 (39.1) | 217 (39.5) |  |
| My mother let me dress in any way I pleased (n=3138) (%) |  |  | 0.357 |
| Moderately like this | 1154 (44.6) | 266 (48.3) |  |
| Moderately unlike this | 583 (22.5) | 123 (22.3) |  |
| Very like this | 551 (21.3) | 102 (18.5) |  |
| Very unlike this | 299 (11.6) | 60 (10.9) |  |
| **Child illness(n=5362) (%)** | 955 (19.9) | 74 (13.4) | <0.001 |
| Child illnesses between age 0 - 60 months (derived from 3 digit ICD codes) (n=5362) (%) |  |  | 0.053 |
| None of the other illnesses | 4305 (89.5) | 531 (96.2) |  |
| Tuberculosis | 22 (0.5) | 1 (0.2) |  |
| Acute specific bacterial and viral childhood infections | 48 (1.0) | 1 (0.2) |  |
| Poliomyelitis | 11 (0.2) | 2 (0.4) |  |
| All other diseases in ICD-8 section I | 38 (0.8) | 0 (0.0) |  |
| Malignant neoplasms | 5 (0.1) | 0 (0.0) |  |
| Benign and unspecified neoplasms | 1 (0.0) | 0 (0.0) |  |
| Endocrine, nutritional and metabolic diseases | 4 (0.1) | 0 (0.0) |  |
| Diseases of blood | 5 (0.1) | 2 (0.4) |  |
| Mental retardation | 50 (1.0) | 1 (0.2) |  |
| Diseases of the central nervous system | 32 (0.7) | 1 (0.2) |  |
| Diseases of the peripheral nervous system | 1 (0.0) | 0 (0.0) |  |
| Diseases of the eye | 1 (0.0) | 0 (0.0) |  |
| Diseases of the ear | 15 (0.3) | 2 (0.4) |  |
| Rheumatic fever | 1 (0.0) | 0 (0.0) |  |
| Other circulatory diseases | 1 (0.0) | 0 (0.0) |  |
| Pneumonia, bronchitis and asthma | 49 (1.0) | 0 (0.0) |  |
| Other respiratory diseases | 10 (0.2) | 0 (0.0) |  |
| Appendicitis | 3 (0.1) | 0 (0.0) |  |
| Other disease of the digestive system | 12 (0.2) | 0 (0.0) |  |
| Genito-urinary system | 12 (0.2) | 2 (0.4) |  |
| Pregnancy and childbirth | 1 (0.0) | 0 (0.0) |  |
| Skin | 4 (0.1) | 0 (0.0) |  |
| Musculo-skeletal | 7 (0.1) | 1 (0.2) |  |
| Congenital | 84 (1.7) | 6 (1.1) |  |
| Early infancy | 52 (1.1) | 0 (0.0) |  |
| Ill-defined | 8 (0.2) | 1 (0.2) |  |
| Accident (traffic) | 5 (0.1) | 1 (0.2) |  |
| Accident (other) | 23 (0.5) | 0 (0.0) |  |
| Child illnesses between age 61-131 months (derived from 3 digit ICD codes) (n=5173) (%) |  |  | 0.505 |
| None of the other illnesses | 4042 (87.5) | 499 (90.4) |  |
| Tuberculosis | 18 (0.4) | 4 (0.7) |  |
| Acute specific bacterial and viral childhood infections | 73 (1.6) | 7 (1.3) |  |
| Poliomyelitis | 22 (0.5) | 3 (0.5) |  |
| All other diseases in ICD-8 section I | 14 (0.3) | 0 (0.0) |  |
| Malignant neoplasms | 3 (0.1) | 0 (0.0) |  |
| Benign and unspecified neoplasms | 1 (0.0) | 0 (0.0) |  |
| Endocrine, nutritional and metabolic diseases | 6 (0.1) | 1 (0.2) |  |
| Diseases of blood | 4 (0.1) | 2 (0.4) |  |
| Mental disorders | 3 (0.1) | 0 (0.0) |  |
| Mental retardation | 47 (1.0) | 1 (0.2) |  |
| Diseases of the central nervous system | 34 (0.7) | 2 (0.4) |  |
| Diseases of the eye | 9 (0.2) | 2 (0.4) |  |
| Diseases of the ear | 18 (0.4) | 2 (0.4) |  |
| Rheumatic fever | 19 (0.4) | 3 (0.5) |  |
| Other circulatory diseases | 2 (0.0) | 0 (0.0) |  |
| Pneumonia and bronchitis, asthma | 52 (1.1) | 5 (0.9) |  |
| Other respiratory diseases | 47 (1.0) | 5 (0.9) |  |
| Appendicitis | 25 (0.5) | 3 (0.5) |  |
| Other disease of the digestive system | 6 (0.1) | 0 (0.0) |  |
| Genito-urinary system | 13 (0.3) | 1 (0.2) |  |
| Pregnancy and childbirth | 1 (0.0) | 0 (0.0) |  |
| Skin | 21 (0.5) | 1 (0.2) |  |
| Musculo-skeletal | 19 (0.4) | 1 (0.2) |  |
| Congenital | 51 (1.1) | 5 (0.9) |  |
| Early infancy | 1 (0.0) | 0 (0.0) |  |
| Ill-defined | 9 (0.2) | 4 (0.7) |  |
| Accident (traffic) | 15 (0.3) | 1 (0.2) |  |
| Accident (other) | 39 (0.8) | 0 (0.0) |  |
| More than one illness period | 7 (0.2) | 0 (0.0) |  |
| Child illnesses between age 132-180 months (derived from 3 digit ICD codes) (n=5165) (%) |  |  | 0.364 |
| None of the other illnesses | 4359 (94.5) | 530 (96.0) |  |
| Tuberculosis | 6 (0.1) | 3 (0.5) |  |
| Poliomyelitis | 10 (0.2) | 4 (0.7) |  |
| All other diseases in ICD-8 section I | 2 (0.0) | 0 (0.0) |  |
| Malignant neoplasms | 2 (0.0) | 0 (0.0) |  |
| Endocrine, nutritional and metabolic diseases | 6 (0.1) | 2 (0.4) |  |
| Diseases of blood | 2 (0.0) | 0 (0.0) |  |
| Mental disorders | 3 (0.1) | 0 (0.0) |  |
| Mental retardation | 50 (1.1) | 1 (0.2) |  |
| Diseases of the central nervous system | 27 (0.6) | 1 (0.2) |  |
| Diseases of the eye | 8 (0.2) | 0 (0.0) |  |
| Diseases of the ear | 8 (0.2) | 1 (0.2) |  |
| Rheumatic fever | 14 (0.3) | 1 (0.2) |  |
| Other circulatory diseases | 1 (0.0) | 0 (0.0) |  |
| Pneumonia and bronchitis, asthma | 18 (0.4) | 2 (0.4) |  |
| Other respiratory diseases | 6 (0.1) | 1 (0.2) |  |
| Appendicitis | 2 (0.0) | 0 (0.0) |  |
| Other disease of the digestive system | 5 (0.1) | 0 (0.0) |  |
| Genito-urinary system | 5 (0.1) | 2 (0.4) |  |
| Skin | 3 (0.1) | 0 (0.0) |  |
| Musculo-skeletal | 14 (0.3) | 1 (0.2) |  |
| Congenital | 38 (0.8) | 2 (0.4) |  |
| Early infancy | 1 (0.0) | 0 (0.0) |  |
| Ill-defined | 2 (0.0) | 0 (0.0) |  |
| Accident (traffic) | 6 (0.1) | 1 (0.2) |  |
| Accident (other) | 15 (0.3) | 0 (0.0) |  |
| **Child maltreatment(n=3040) (%)** | 153 (6.1) | 36 (6.5) | 0.818 |
| As a child do you feel you were mistreated by your parents in any way? (n=3040) = Yes (%) | 153 (6.1) | 36 (6.5) | 0.818 |
| **Adverse Childhood Experiences (ACE) count score(n=2549) (%)** |  |  | 0.232 |
| 0 | 923 (46.2) | 232 (42.0) |  |
| 1 | 710 (35.6) | 227 (41.1) |  |
| 2 | 279 (14.0) | 70 (12.7) |  |
| 3 | 72 (3.6) | 19 (3.4) |  |
| 4 | 12 (0.6) | 3 (0.5) |  |
| 5 | 1 (0.1) | 1 (0.2) |  |

**Supplementary Table 4** *Associations of the ACE score with depression in the full cohort of ALSPAC women (N=5,590), and the subsample included in our analysis (N=700). Analyses are restricted to women with complete data on the ACE score and depression, assessed as a score of 13 or more on the Edinburgh Postnatal Depression score, which was completed in a questionnaire when the woman’s child was aged 11 years.*

|  | Odds ratio (95% CI) for depression | |
| --- | --- | --- |
|  | Full cohort (N=5,590) | Women included in our analysis (N=700) |
| ACE score |  |  |
| 0 | 1 (ref) | 1 (ref) |
| 1 | 1.2 (0.9 to 1.4) | 1.5 (0.9 to 2.7) |
| 2 | 1.6 (1.3 to 2.1) | 2.0 (1.0 to 4.0) |
| 3 | 2.1 (1.6 to 2.9) | 1.2 (0.5 to 3.4) |
| 4 or more | 3.8 (2.9 to 5.0) | 4.9 (2.4 to 9.9) |

***Supplementary Table 5*** *Comparison of included (n=98) and excluded participants for the tissue comparison analysis in the NSHD cohort. Participants were excluded if they had missing data on one of the adversity measures, no buccal DNA methylation measured or no whole blood DNA methylation measured.*

| **Description (n with available data)** | **Excluded** | **Included** | **p** (Fisher exact test) |
| --- | --- | --- | --- |
| Total sample size (n=552) | 454 | 98 |  |
| Sex(n=552) = Female (%) | 454 (100.0) | 98 (100.0) | NA |
| Age clinic(n=552) (mean (sd)) | 53.46 (0.50) | 53.41 (0.49) | 0.327 |
| Smoking - history up to 53 years(n=552) (%) |  |  | 0.621 |
| Never-smoker | 149 (32.8) | 35 (35.7) |  |
| Ex-smoker | 219 (48.2) | 42 (42.9) |  |
| Current-smoker | 86 (18.9) | 21 (21.4) |  |
| **Parent physically ill(n=552) (%)** | 121 (26.7) | 22 (22.4) | 0.463 |
| **Parent mentally ill(n=552) (%)** | 10 (2.2) | 0 (0.0) | 0.287 |
| **Parents separated(n=552) (%)** | 18 (4.0) | 5 (5.1) | 0.816 |
| **Parent died(n=552) (%)** | 26 (5.7) | 9 (9.2) | 0.296 |
| **Sub optimal maternal bonding(n=552) (%)** | 104 (22.9) | 16 (16.3) | 0.194 |
| **Child illness(n=552) (%)** | 63 (13.9) | 11 (11.2) | 0.592 |
| **Child maltreatment(n=552) (%)** | 31 (6.8) | 5 (5.1) | 0.688 |
| **Adverse Childhood Experiences count score(n=552) (%)** |  |  | 0.148 |
| 0 | 185 (40.7) | 47 (48.0) |  |
| 1 | 188 (41.4) | 39 (39.8) |  |
| 2 | 61 (13.4) | 9 (9.2) |  |
| 3 | 17 (3.7) | 2 (2.0) |  |
| 4 | 3 (0.7) | 0 (0.0) |  |
| 5 | 0 (0.0) | 1 (1.0) |  |


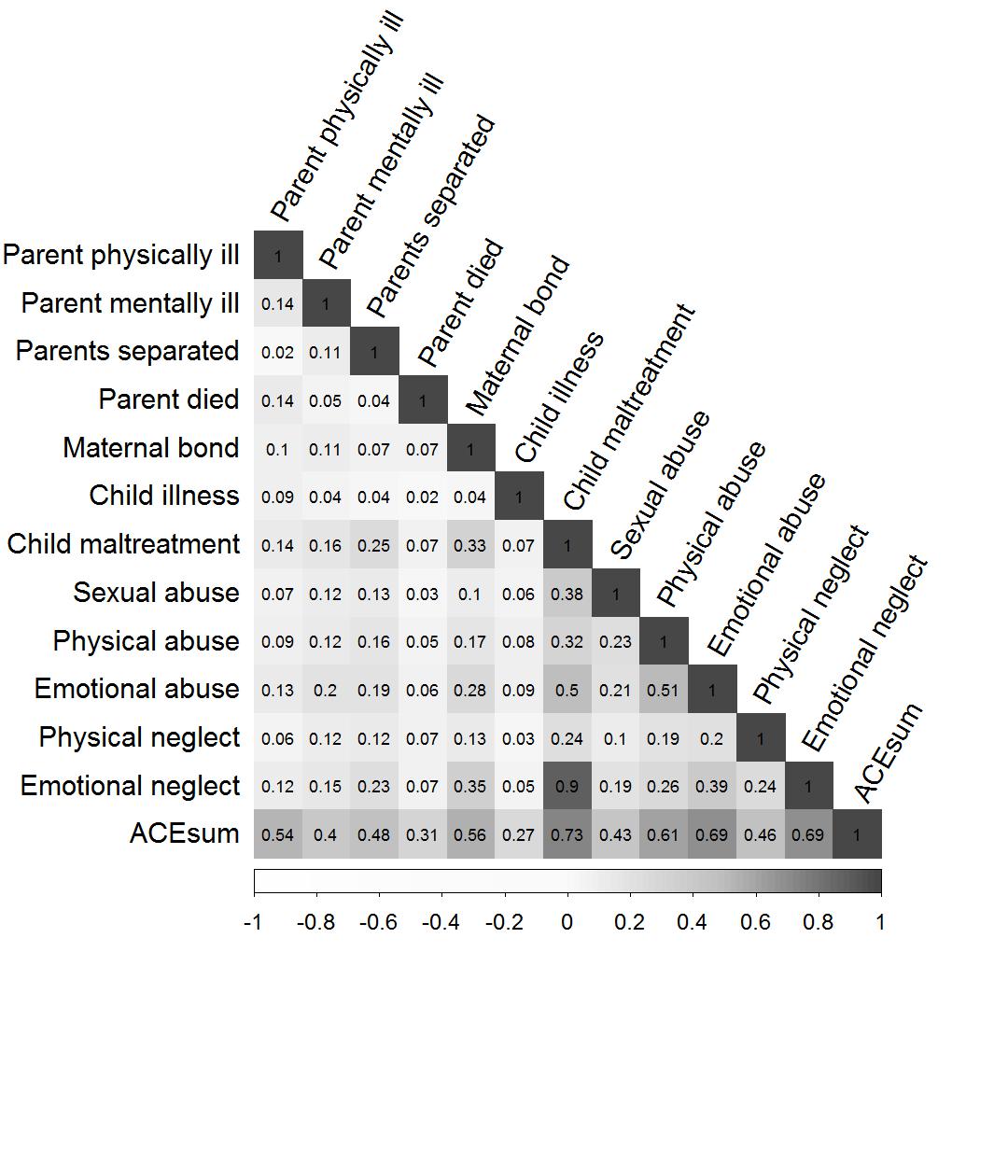


**Supplementary Figure 3**. *Correlation (Cramer V based on chi squared) between the childhood adversity measures in ALSPAC (n=780). The shading indicates the strength of the correlation from low (white) to high (dark grey).*

*
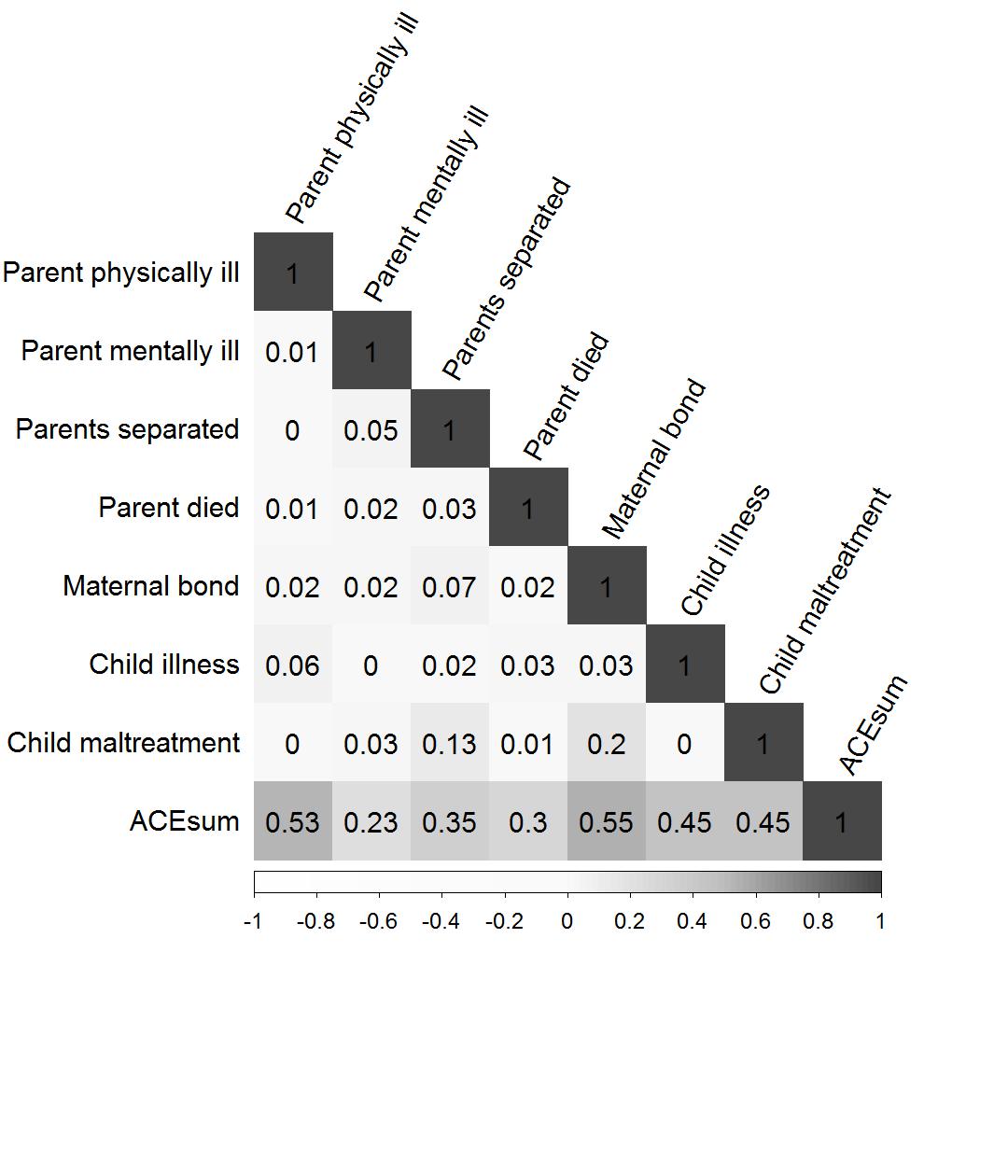
*

**Supplementary Figure 4**. *Correlation (Cramer V based on chi squared) between the childhood adversity measures in NSHD (n=552). The shading indicates the strength of the correlation from low (white) to high (dark grey).*

***
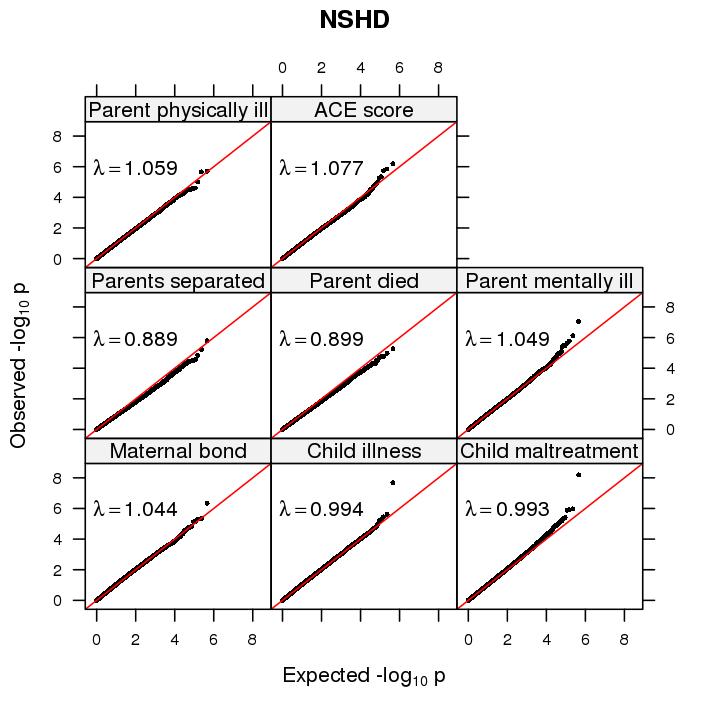
***

**Supplementary Figure 5**. *Overview of quantile-quantile plots to compare the obtained p-value distribution to the expected p-value distribution for each childhood adversity exposure examined with an epigenome wide analysis (EWAS) in the NSHD cohort.*

***
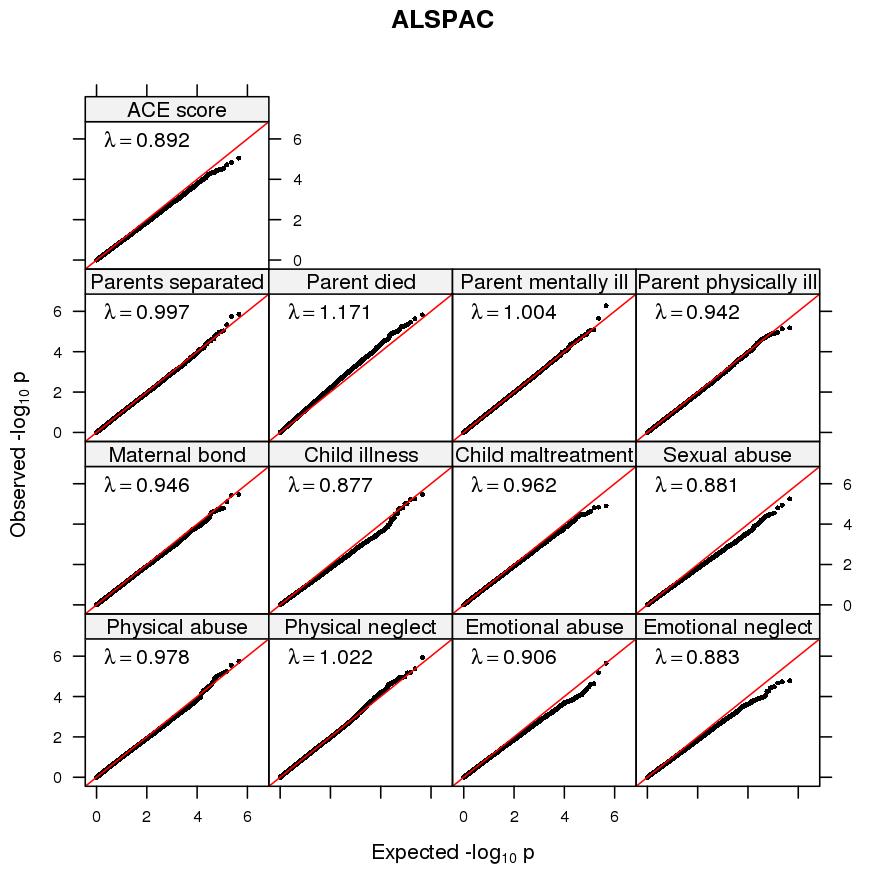
*Supplementary Figure 6**. *Overview of quantile-quantile plots to compare the obtained p-value distribution to the expected p-value distribution for each childhood adversity exposure examined with an epigenome wide analysis (EWAS) in the ALSPAC cohort.*

***Enrichment for probes previously reported in literature***

A PubMed search on February 2018 with the keywords [(early life OR childhood OR child) AND (adversity OR trauma OR maltreatment OR stress) AND (epigenetic OR methylation)] identified 1035 publications of interest. After excluding publications that did not describe new results (n=632), did not measure methylation using the Infinium HumanMethylation450BeadChip (Illumina, Inc) (n=190), were performed in non-human subjects (n=196), examined non-ACE exposures (prenatal adversity, caffeine consumption, atherosclerosis, preterm birth, asthma) (n=5), examined other outcomes (depression) (n=1) or did not report the Illumina CpG identifiers (n=2), nine studies were examined for enrichment (see Supplementary Table 5).

**
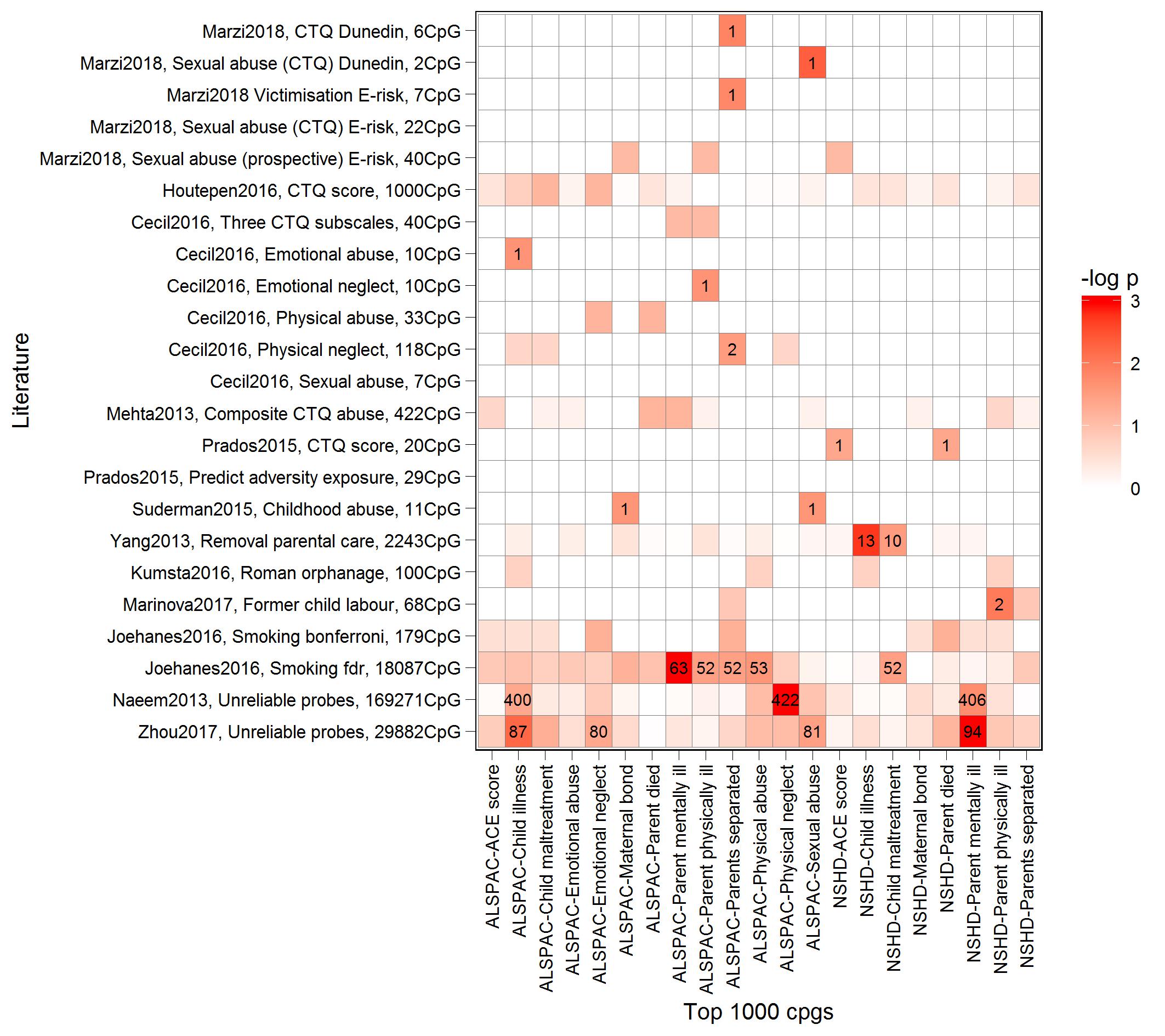
**

**Supplementary Figure 7** *Enrichment of CpGs from literature (y-axis) in the top 1000 CpGs of the childhood adversity analyses (x-axis). If there was significant enrichment (p<0.05 in a Fisher exact test), the number of overlapping CpGs is given. A darker colour intensity indicates a lower p-value on the Fisher exact test. Abbreviations: CTQ = childhood trauma questionnaire.*

***Supplementary Table 6*** *Details of the nine studies reporting CpGs measured on the Illumina Infinium HumanMethylation450 BeadChip that associated with childhood adversity.*

| **Article** | **Childhood adversity** | **Population** | **Tissue** | **Nr CpGs** | **CpG selection criteria** |
| --- | --- | --- | --- | --- | --- |
| (Suderman et al., 2015) | Childhood abuse | 12 cases – 28 control males from the 1958 British Birth Cohort | Whole blood | 11 | FDR < 0.2 |
| (Yang et al., 2013) | Removal parental care | 96 cases – 96 controls | Saliva | 2868 | p < 5.0 x 10^-07^ |
| (Houtepen et al., 2016) | Total score on the childhood trauma questionnaire (CTQ) | 89 healthy controls | Whole blood | 1000 | Top CpGs |
| (Marzi et al., 2018) | Multiple forms of victimization in childhood and adolescence (including physical, sexual, and emotional abuse; neglect; intimate-partner violence; bullying; cyber-victimization; and crime) | 1,658 participants (including 428 monozygotic twin pairs and 306 dizygotic twin pairs) | Blood | 7 | Array threshold p < 1.16 x 10^-07^ |
|  | Sexual abuse either 0-12 years or 12-16 years, prospectively reported |  |  | 40 |  |
|  | Sexual abuse 0-12 years, retrospectively reported on the CTQ |  |  | 22 |  |
|  | Sexual abuse 0-16 years, retrospectively reported on the CTQ | 818 Dunedin study members | Blood | 2 |  |
|  | Childhood adversity 0-16 years, retrospectively reported on the CTQ |  |  | 6 |  |
| (Kumsta et al., 2016) | Institutionalised Romanian orphanage | 33 Romanian cases – 16 UK control adoptees | Buccal | 100 | Top CpGs |
| (Prados et al., 2015) | CTQ score | 96 borderline case – 93 depressive controls | Blood leukocytes | 31 | Predict adversity exposure |
|  |  |  |  | 20 | Top correlated CpGs |
| (Cecil et al., 2016) | Sexual abuse subscale on CTQ | 124 high-risk sample of inner-city youth | Buccal | 7 | FDR < 0.05 |
|  | Physical abuse subscale on CTQ |  |  | 34 | FDR < 0.05 |
|  | Physical neglect subscale on CTQ |  |  | 118 | FDR < 0.05 |
|  | Emotional abuse subscale on CTQ |  |  | 10 | Top CpGs |
|  | Emotional neglect subscale on CTQ |  |  | 10 | Top CpGs |
|  | Sexual abuse, physical abuse and physical neglect subscales on CTQ |  |  | 40 | Top 20 either positively or negatively associated with all three adversities |
| (Mehta et al., 2013) | Composite abuse variable based on cut-off for sexual, physical, and emotional abuse subscales on CTQ | 61 posttraumatic stress disorder cases (32 exposed and 29 non-exposed to childhood abuse) – 108 trauma exposed controls | Blood | 447 | Near genes with childhood trauma associated gene expression differences |
| (Marinova et al., 2017) | Former child labour (checked with score on the CTQ – short form) | 30 former indentured child labourers – 15 demographically matched controls | Buccal | 71 | FDR < 0.05 |

**References for the studies in Supplementary Table 6**

Cecil, C. A. M., Smith, R. G., Walton, E., Mill, J., McCrory, E. J., & Viding, E. (2016). Epigenetic signatures of childhood abuse and neglect: Implications for psychiatric vulnerability. *Journal of Psychiatric Research*, *83*, 184–194. http://doi.org/10.1016/j.jpsychires.2016.09.010

Houtepen, L. C., Vinkers, C. H., Carrillo-Roa, T., Hiemstra, M., van Lier, P. A., Meeus, W., … Boks, M. P. M. (2016). Genome-wide DNA methylation levels and altered cortisol stress reactivity following childhood trauma in humans. *Nature Communications*, *7*, 10967. http://doi.org/10.1038/ncomms10967

Kumsta, R., Marzi, S. J., Viana, J., Dempster, E. L., Crawford, B., Rutter, M., … Sonuga-Barke, E. J. S. (2016). Severe psychosocial deprivation in early childhood is associated with increased DNA methylation across a region spanning the transcription start site of CYP2E1. *Translational Psychiatry*, *6*(6), e830. http://doi.org/10.1038/tp.2016.95

Marinova, Z., Maercker, A., Kuffer, A., Robinson, M. D., Wojdacz, T. K., Walitza, S., … Burri, A. (2017). DNA methylation profiles of elderly individuals subjected to indentured childhood labor and trauma. *BMC Med Genet*, *18*(1), 21. http://doi.org/10.1186/s12881-017-0370-2

Marzi, S. J., Sugden, K., Arseneault, L., Belsky, D. W., Burrage, J., Corcoran, D. L., … Caspi, A. (2018). Analysis of DNA Methylation in Young People: Limited Evidence for an Association Between Victimization Stress and Epigenetic Variation in Blood. *American Journal of Psychiatry*, appi.ajp.2017.17060693. http://doi.org/10.1176/appi.ajp.2017.17060693

Mehta, D., Klengel, T., Conneely, K. N., Smith, A. K., Altmann, A., Pace, T. W., … Binder, E. B. (2013). Childhood maltreatment is associated with distinct genomic and epigenetic profiles in posttraumatic stress disorder. *Proc.Natl.Acad.Sci.U.S.A*, *110*(1091–6490 (Electronic)), 8302–8307.

Prados, J., Stenz, L., Courtet, P., Prada, P., Nicastro, R., Adouan, W., … Perroud, N. (2015). Borderline personality disorder and childhood maltreatment: A genome-wide methylation analysis. *Genes, Brain and Behavior*, *14*(2), 177–188. http://doi.org/10.1111/gbb.12197

Suderman, M., Pappas, J. J., Borghol, N., Buxton, J. L., McArdle, W. L., Ring, S. M., … Pembrey, M. (2015). Lymphoblastoid cell lines reveal associations of adult DNA methylation with childhood and current adversity that are distinct from whole blood associations. *International Journal of Epidemiology*, *44*(4), 1331–1340. http://doi.org/10.1093/ije/dyv168

Yang, B.-Z., Zhang, H., Ge, W., Weder, N., Douglas-Palumberi, H., Perepletchikova, F., … Kaufman, J. (2013). Child abuse and epigenetic mechanisms of disease risk. *American Journal of Preventive Medicine*, *44*(2), 101–7. http://doi.org/10.1016/j.amepre.2012.10.012

***Supplementary Table 7*** *The DMRs for the ACE measures available in both cohorts.*

| **Discovery EWAS** | **Nr DMR Šidák <0.05** | **Nr replicated DMRs** | **DMR** | **Nr CpGs** | **Gene** | **Direction of effect each CpG** | **P region** | **P Šidák** | **Look up ALSPAC**  (Direction^1^, p-value region) | **Look up NSHD** (Direction^1^, p-value region) | **Look up NSHD blood**^2^ (Direction^1^, p-value region) |
| --- | --- | --- | --- | --- | --- | --- | --- | --- | --- | --- | --- |
| NSHD- ACE score | 18 | 1 | chr8: 145654565-145654855 | 5 | *VPS28, TONSL* | ----- | 1.8E-06 | 2.9E-03 | **ACE score: -----, p=7.5e-08*** Parent mentally ill: -----, p=0.00025* Parent physically ill: -----, p=0.035* Parent died: -----, p=0.049* Parents separated: -----, p=4.9e-06* Maternal bond: -----, p=2.6e-05* Child illness: ++---, p=0.98 Child maltreatment -----, p=0.019* Physical abuse: -----, p=0.51 Physical neglect: -----, p=0.0065* Sexual abuse: -----, p=0.68 Emotional abuse: -----, p=8.9e-05* Emotional neglect: -----, p=0.02* ACE score smoking: -----, p=3e-11* | **ACE score: -----, p=1.8e-06*** Parent mentally ill: ++---, p=0.64 Parent physically ill: ----+, p=0.8 Parent died: -----, p=0.012* Parents separated: +++++, p=0.52 Maternal bond: --+--, p=0.036* Child illness: -----, p=4.2e-08* Child maltreatment ----+, p=0.013* ACE score smoking: -----, p=0.00011* | **ACEsum: -----, p= 0.05***  Parent physically ill: -----, p= 0.00068*  Parent died: -----, p= 0.44  Parents separated: +++++, p= 0.044*  Maternal bond: +-++-, p= 0.98  Child illness: -----, p= 0.57  Child maltreatment: -----, p= 0.49 |
| NSHD- Child illness | 18 | 0 | n/a | n/a | n/a | n/a | n/a | n/a | n/a | n/a | n/a |
| NSHD- Child maltreatment | 29 | 0 | n/a | n/a | n/a | n/a | n/a | n/a | n/a | n/a | n/a |
| NSHD- Maternal bond | 13 | 0 | n/a | n/a | n/a | n/a | n/a | n/a | n/a | n/a | n/a |
| NSHD- Parent died | 6 | 0 | n/a | n/a | n/a | n/a | n/a | n/a | n/a | n/a | n/a |
| NSHD- Parent mentally ill | 14 | 2 | chr12: 14720726-14721289 | 10 | *PLBD1* | +++++++++- | 9.5E-07 | 7.7E-04 | ACE score: -+++-+++++, p=0.86 **Parent mentally ill: -++-++++++, p=0.0058*** Parent physically ill: ---+----++, p=0.59 Parent died: --+-++-+++, p=0.95 Parents separated: -++++++++-, p=5.3e-05* Maternal bond: ---+-++++-, p=0.77 Child illness: ----++++++, p=0.7 Child maltreatment ++++----+-, p=0.56 Physical abuse: +--------+, p=0.26 Physical neglect: +-----+---, p=0.88 Sexual abuse: +-++-----+, p=0.0015* Emotional abuse: +--+----+-, p=0.6 Emotional neglect: -+++------, p=0.78 Parent mentally ill smoking: -++-++++++, p= 0.0088* | ACE score: ++++++++++, p=0.32 **Parent mentally ill: +++++++++-, p=9.5e-07*** Parent physically ill: +++++++--+, p=0.18 Parent died: --+-----++, p=0.93 Parents separated: --++-+--++, p=0.25 Maternal bond: -------+++, p=0.25 Child illness: ++++++++++, p=0.76 Child maltreatment +----+-+++, p=0.74 Parent mentally ill smoking: ++++++++++, p=1.8e-08* | ACEsum: -+---++-++, p= 0.88  **Parent mentally ill: n/a**  ACEbin: -----+++++, p= 0.36  Parent physically ill: ++-------+, p= 0.084  Parent died: --+-+-++++, p= 0.81  Parents separated: +--+--++--, p= 0.85  Maternal bond: ------+-++, p= 0.23  Child illness: ---------+, p= 0.17  Child maltreatment: -+-+-+--++, p= 0.7 |
|  |  |  | chr1: 3104999-3105327 | 5 | *PRDM16* | ----- | 7.4E-08 | 1.0E-04 | ACE score: ++++-, p=0.77 **Parent mentally ill: -----, p=0.001*** Parent physically ill: -+--+, p=0.74 Parent died: +++++, p=0.074 Parents separated: +-+--, p=0.96 Maternal bond: ++--+, p=0.98 Child illness: -+---, p=0.9 Child maltreatment ++++-, p=0.009* Physical abuse: +++++, p=0.083 Physical neglect: --++-, p=0.94 Sexual abuse: ++-++, p=0.84 Emotional abuse: ++++-, p=0.3 Emotional neglect: ++++-, p=0.33 Parent mentally ill smoking: -----, p= 5.3e-05* | ACE score: +++++, p=0.4 **Parent mentally ill: -----, p=7.4e-08*** Parent physically ill: +++-+, p=0.78 Parent died: ++++-, p=0.98 Parents separated: ----+, p=0.13 Maternal bond: +++++, p=0.25 Child illness: ++++-, p=0.0035* Child maltreatment --++-, p=0.9 Parent mentally ill smoking: -----, p=2.2e-06* | ACEsum: +-+-+, p= 0.95 **Parent mentally ill: n/a**  ACEbin: +-++-, p= 0.98  Parent physically ill: ++++-, p= 0.73  Parent died: --+++, p= 0.98  Parents separated: +++++, p= 0.45  Maternal bond: +++++, p= 0.52  Child illness: -----, p= 0.71  Child maltreatment: -----, p= 0.24 |
| NSHD- Parent physically ill | 23 | 1 | chr15: 81426347-81426670 | 9 | *C15orf26* | +++++++++ | 7.4E-07 | 1.0E-03 | ACE score: -+++----+, p=0.98 Parent mentally ill: +++++----, p=0.94 **Parent physically ill: +++++++++, p=0.011*** Parent died: --+++++++, p=0.72 Parents separated: ---------, p=0.036* Maternal bond: +++--+---, p=0.78 Child illness: --+--+-++, p=0.9 Child maltreatment -+-+-+---, p=0.83 Physical abuse: -+-+-+---, p=0.84 Physical neglect: ---------, p=0.6 Sexual abuse: ---------, p=0.28 Emotional abuse: -++------, p=0.87 Emotional neglect: -+++++-++, p=0.92 Parent physically ill smoking: +++++++++, p= 0.00087* | ACE score: +++++++++, p=7.1e-09* Parent mentally ill: ++-++++++, p=0.71 **Parent physically ill: +++++++++, p=7.4e-07*** Parent died: +++++++++, p=0.16 Parents separated: +-+++++-+, p=0.98 Maternal bond: --+--++++, p=0.93 Child illness: ++-++++++, p=0.13 Child maltreatment +++++++++, p=0.016* Parent physically ill smoking: +++++++++, p=1e-07* | ACEsum: +++++++++, p= 0.29  **Parent physically ill: ++-+-+++-, p= 0.99**  Parent died: -+-+-++++, p= 0.98  Parents separated: ---------, p= 0.83  Maternal bond: +++-+--+-, p= 0.99  Child illness: ---------, p= 0.00018*  Child maltreatment: +++++++++, p= 0.052 |
| NSHD- Parents separated | 13 | 0 | n/a | n/a | n/a | n/a | n/a | n/a | n/a | n/a | n/a |
| ALSPAC- ACE score | 8 | 1 | chr8: 145654565-145654855 | 5 | *VPS28, TONSL* | ----- | 7.5E-08 | 1.2E-04 | **ACE score: -----, p=7.5e-08*** Parent mentally ill: -----, p=0.00025* Parent physically ill: -----, p=0.035* Parent died: -----, p=0.049* Parents separated: -----, p=4.9e-06* Maternal bond: -----, p=2.6e-05* Child illness: ++---, p=0.98 Child maltreatment -----, p=0.019* Physical abuse: -----, p=0.51 Physical neglect: -----, p=0.0065* Sexual abuse: -----, p=0.68 Emotional abuse: -----, p=8.9e-05* Emotional neglect: -----, p=0.02* ACE score smoking: -----, p=3e-11* | **ACE score: -----, p=1.8e-06*** Parent mentally ill: ++---, p=0.64 Parent physically ill: ----+, p=0.8 Parent died: -----, p=0.012* Parents separated: +++++, p=0.52 Maternal bond: --+--, p=0.036* Child illness: -----, p=4.2e-08* Child maltreatment ----+, p=0.013* ACE score smoking: -----, p=0.00011* | **ACEsum: -----, p= 0.05***  Parent physically ill: -----, p= 0.00068*  Parent died: -----, p= 0.44  Parents separated: +++++, p= 0.044*  Maternal bond: +-++-, p= 0.98  Child illness: -----, p= 0.57  Child maltreatment: -----, p= 0.49 |
| ALSPAC- Child illness | 3 | 0 | n/a | n/a | n/a | n/a | n/a | n/a | n/a | n/a | n/a |
| ALSPAC- Child maltreatment | 7 | 0 | n/a | n/a | n/a | n/a | n/a | n/a | n/a | n/a | n/a |
| ALSPAC- Maternal bond | 4 | 0 | n/a | n/a | n/a | n/a | n/a | n/a | n/a | n/a | n/a |
| ALSPAC- Parent died | 20 | 3 | chr15: 40364524-40364863 | 3 |  | +++ | 6.3E-07 | 8.4E-04 | ACE score: +++, p=3e-04* Parent mentally ill: +++, p=0.014* Parent physically ill: +++, p=0.21 **Parent died: +++, p=6.3e-07*** Parents separated: +++, p=0.22 Maternal bond: +++, p=0.46 Child illness: +--, p=0.75 Child maltreatment +++, p=0.22 Physical abuse: -++, p=0.89 Physical neglect: +++, p=0.13 Sexual abuse: +++, p=0.31 Emotional abuse: +++, p=0.0034* Emotional neglect: +++, p=0.13 Parent died smoking: +++, p=1.5e-06* | ACE score: -++, p=0.84 Parent mentally ill: +++, p=0.048* Parent physically ill: +++, p=0.51 **Parent died: +++, p=0.0044*** Parents separated: +++, p=0.42 Maternal bond: ---, p=0.61 Child illness: ---, p=0.01* Child maltreatment -++, p=0.81 Parent died smoking: +++, p= 0.0032* | ACEsum: +++, p= 0.89  Parent physically ill: +++, p= 0.2  **Parent died: +++, p= 0.73**  Parents separated: ---, p= 0.7  Maternal bond: +--, p= 0.99  Child illness: ---, p= 0.00057*  Child maltreatment: +++, p= 0.29 |
|  |  |  | chr7: 24323261-24323940 | 9 | *NPY* | +++++++++ | 9.2E-07 | 6.2E-04 | ACE score: ++++++-++, p=0.52 Parent mentally ill: --+++++++, p=0.09 Parent physically ill: ++++++++-, p=0.7 **Parent died: +++++++++, p=9.2e-07*** Parents separated: -+++++-++, p=0.79 Maternal bond: +++++++-+, p=0.068 Child illness: -++---++-, p=0.62 Child maltreatment -+-+-----, p=0.65 Physical abuse: ++-++---+, p=0.68 Physical neglect: --+--++--, p=0.81 Sexual abuse: +++-++++-, p=0.92 Emotional abuse: --+++---+, p=0.69 Emotional neglect: -+++---++, p=0.9 Parent died smoking: +++++++++, p=0.00019* | ACE score: --+--+---, p=0.79 Parent mentally ill: +-++++-++, p=0.99 Parent physically ill: --+------, p=0.2 **Parent died: +++++++++, p=0.014*** Parents separated: +++--+---, p=0.73 Maternal bond: +++-+-++-, p=0.96 Child illness: -+---+---, p=0.1 Child maltreatment -----+---, p=0.13  Parent died smoking: +-+++++++, p= 0.11 | ACEsum: ++---++-+, p= 0.94  Parent physically ill: ------+--, p= 0.95  **Parent died: +++++---+, p= 0.34**  Parents separated: --++-++-+, p= 0.89  Maternal bond: ++-++--++, p= 0.91  Child illness: ------++-, p= 0.99  Child maltreatment: +-+++++++, p= 0.75 |
|  |  |  | chr2: 18766018-18766295 | 4 | *NT5C1B* | ---+ | 9.8E-06 | 1.6E-02 | ACE score: ---+, p=0.92 Parent mentally ill: +--+, p=0.83 Parent physically ill: ++-+, p=0.68 **Parent died: ---+, p=9.8e-06*** Parents separated: ---+, p=0.35 Maternal bond: ---+, p=0.95 Child illness: +++-, p=0.23 Child maltreatment ++++, p=0.054 Physical abuse: ----, p=0.89 Physical neglect: ---+, p=0.3 Sexual abuse: +-++, p=0.77 Emotional abuse: ++++, p=0.15 Emotional neglect: ++++, p=0.16 Parent died smoking: ---+, p=7.5e-06* | ACE score: +---, p=0.86 Parent mentally ill: ----, p=0.19 Parent physically ill: +++-, p=0.67 **Parent died: ----, p=0.014*** Parents separated: ++++, p=0.87 Maternal bond: +-++, p=0.78 Child illness: ---+, p=0.91 Child maltreatment +-+-, p=0.84  Parent died smoking: ----, p= 0.046* | ACEsum: +--+, p= 0.82  Parent physically ill: +++-, p= 0.17  **Parent died: ---+, p= 0.0047***  Parents separated: ----, p= 0.44  Maternal bond: ----, p= 0.017*  Child illness: ----, p= 0.3  Child maltreatment: --+-, p= 0.89 |
| ALSPAC- Parent mentally ill | 10 | 0 | n/a | n/a | n/a | n/a | n/a | n/a | n/a | n/a | n/a |
| ALSPAC- Parent physically ill | 14 | 2 | chr22: 27834439-27834630 | 3 |  | --- | 6.9E-06 | 1.6E-02 | ACE score: -++, p=0.81 Parent mentally ill: +++, p=0.029* **Parent physically ill: ---, p=6.9e-06*** Parent died: +++, p=0.28 Parents separated: --+, p=0.24 Maternal bond: -++, p=0.81 Child illness: +++, p=0.92 Child maltreatment ---, p=0.83 Physical abuse: +-+, p=0.92 Physical neglect: ---, p=0.89 Sexual abuse: -++, p=0.97 Emotional abuse: -++, p=0.71 Emotional neglect: -++, p=0.69 Parent physically ill smoking: ---, p=0.00027* | ACE score: +++, p=0.93 Parent mentally ill: ---, p=0.59 **Parent physically ill: ---, p=0.045*** Parent died: -+-, p=1 Parents separated: +++, p=0.23 Maternal bond: --+, p=0.91 Child illness: +++, p=0.0025* Child maltreatment +++, p=0.69 Parent physically ill smoking: ---, p= 0.063 | ACEsum: --+, p= 0.92  **Parent physically ill: +++, p= 0.39**  Parent died: +++, p= 0.93  Parents separated: +++, p= 0.03*  Maternal bond: ---, p= 0.38  Child illness: +-+, p= 0.75  Child maltreatment: ---, p= 0.0023* |
|  |  |  | chr8: 144120335-144120707 | 7 | *C8orf31* | +++++++ | 4.4E-06 | 5.4E-03 | ACE score: +++++++, p=0.0035* Parent mentally ill: --+++++, p=0.96 **Parent physically ill: +++++++, p=4.4e-06*** Parent died: +++++++, p=0.0098* Parents separated: -------, p=0.14 Maternal bond: +++++++, p=0.68 Child illness: +++++++, p=0.0024* Child maltreatment -+-+--+, p=0.97 Physical abuse: ++-++++, p=0.81 Physical neglect: +--++-+, p=0.9 Sexual abuse: --+----, p=0.4 Emotional abuse: +++++++, p=0.086 Emotional neglect: ++++-++, p=0.96 Parent physically ill smoking: +++++++, p=0.00025* | ACE score: +++++++, p=0.4 Parent mentally ill: --+----, p=0.75 **Parent physically ill: +++++++, p=0.00092*** Parent died: +++++++, p=0.00035* Parents separated: -------, p=3.8e-05* Maternal bond: -+++-+-, p=0.97 Child illness: --+----, p=0.81 Child maltreatment --++-+-, p=0.88 Parent physically ill smoking: +++++++, p= 0.058 | ACEsum: +++++++, p= 1.3e-05*  ACEbin: +-+++++, p= 0.85  Parent physically ill: +++++++, p= 0.13  Parent died: +-+++++, p= 0.75  Parents separated: -------, p= 0.87  Maternal bond: ----+++, p= 0.93  Child illness: +++++++, p= 0.082  Child maltreatment: -------, p= 0.00076* |
| ALSPAC- Parents separated | 10 | 0 | n/a | n/a | n/a | n/a | n/a | n/a | n/a | n/a | n/a |

*The first three columns indicate how many of the DMRs with Šidák corrected p-value <0.05 in the original analysis replicate (same direction, same exposure) in the other cohort, while the last eight columns give information on these replicated DMRs.*

** in the last two columns indicates a nominal significant association (p-value region <0.05) between the DMR and one of the childhood adversities in the respective cohort, NSHD or ALSPAC.*

*^1^* *Direction of effect for each individual CpG that is part of the DMR was derived from the regression coefficient in the epigenome wide analysis for individual CpGs.*

*^2^ It was not possible to look at parental mental illness in the smaller NSHD subset with DNA methylation measurement in both whole blood and buccal tissue, due to low prevalence numbers.*

***Supplementary Table 8*** *The DMRs for the abuse and neglect measures in ALSPAC.*

| **Discovery EWAS** | **Nr DMR Šidák <0.05** | **DMR** | **Nr CpGs** | **Gene** | **Direction of effect each CpG** | **P region** | **P Šidák** | **Look up NSHD** (Direction, p-value region) | **Look up ALSPAC**  (Direction, p-value region) |
| --- | --- | --- | --- | --- | --- | --- | --- | --- | --- |
| ALSPAC- Emotional abuse | 3 | chr1: 59042931-59043371 | 9 | *TACSTD2* | +++++++++ | 1.33E-07 | 0.000137 | ACE score: ++----+--, p=0.9 Parent mentally ill: +--------, p=0.37 Parent physically ill: +++++++++, p=0.68 Parent died: +++++++++, p=0.57 Parents separated: ---------, p=0.73 Maternal bond: +--------, p=0.34 Child illness: ------+--, p=0.72 Child maltreatment: +++--++-+, p=0.97 | ACE score: +++++++++, p=0.0011* Parent mentally ill: ---------, p=0.092 Parent physically ill: ++-++++++, p=0.083 Parent died: ++++--+++, p=0.92 Parents separated: +++++++++, p=0.0034* Maternal bond: +++++-+-+, p=0.81 Child illness: ---+--+-+, p=0.95 Child maltreatment: +++++++++, p=0.00091* Physical abuse: ---------, p=0.95 Physical neglect: --------+, p=0.99 Sexual abuse: +++++++++, p=0.0024* **Emotional abuse: +++++++++, p=1.3e-07*** Emotional neglect: +++++++++, p=0.024* Emotional abuse smoking: +++++++++, p=7e-10* |
|  |  | chr1: 175856781-175856914 | 2 |  | -- | 7.06E-07 | 0.002405 | ACE score: +-, p=0.82 Parent mentally ill: +-, p=0.81 Parent physically ill: --, p=0.64 Parent died: ++, p=0.97 Parents separated: --, p=0.98 Maternal bond: ++, p=0.31 Child illness: +-, p=0.44 Child maltreatment: --, p=0.67 | ACE score: --, p=0.0062* Parent mentally ill: -+, p=0.84 Parent physically ill: --, p=0.51 Parent died: ++, p=0.36 Parents separated: -+, p=0.7 Maternal bond: --, p=0.011* Child illness: ++, p=0.11 Child maltreatment: --, p=0.0016* Physical abuse: --, p=0.27 Physical neglect: --, p=0.46 Sexual abuse: +-, p=0.44 **Emotional abuse: --, p=7.1e-07*** Emotional neglect: --, p=0.054 Emotional abuse smoking: --, p=1.1e-06* |
|  |  | chr11: 67417958-67418406 | 13 | *ACY3* | ------------- | 7.62E-08 | 7.72E-05 | ACE score: +++++--++-+-+, p=0.98 Parent mentally ill: ++++++++++++-, p=0.097 Parent physically ill: +-+---------+, p=0.73 Parent died: -+-+-++-----+, p=0.95 Parents separated: --+++-+++++-+, p=0.95 Maternal bond: +-++---+++--+, p=0.97 Child illness: +++----+----+, p=0.96 Child maltreatment: ++++++++++++-, p=0.0039* | ACE score: -------------, p=4.4e-05* Parent mentally ill: ++--+-+---++-, p=0.97 Parent physically ill: --+++++--+-+-, p=0.93 Parent died: -+++++++++++-, p=0.14 Parents separated: ---+----+--+-, p=0.8 Maternal bond: -------------, p=0.026* Child illness: +++++-+---+++, p=0.99 Child maltreatment: -------------, p=6e-13* Physical abuse: -------------, p=0.0021* Physical neglect: -+-----+-----, p=0.83 Sexual abuse: ----------+--, p=0.2 **Emotional abuse: -------------, p=7.6e-08*** Emotional neglect: -------------, p=2.1e-10* Emotional abuse smoking: -------------, p=2e-07*  Emotional neglect smoking: -------------, p=3.9e-08* |
| ALSPAC- Emotional neglect | 5 | chr6: 166259938-166260573 | 6 |  | ------- | 1.50E-08 | 1.07E-05 | ACE score: -----+-, p=0.58 Parent mentally ill: ++++---, p=0.31 Parent physically ill: -------, p=0.0054* Parent died: ++++--+, p=0.9 Parents separated: -+-++++, p=0.6 Maternal bond: ++++++-, p=0.64 Child illness: --++++-, p=0.81 Child maltreatment: +-+----, p=0.7 | ACE score: -------, p=3e-05* Parent mentally ill: --++-+-, p=0.64 Parent physically ill: --++---, p=0.95 Parent died: +++----, p=0.69 Parents separated: -----+-, p=0.5 Maternal bond: -----+-, p=0.1 Child illness: ++++---, p=0.87 Child maltreatment: -----+-, p=0.0002* Physical abuse: -------, p=0.36 Physical neglect: +--++--, p=0.71 Sexual abuse: -----+-, p=0.091 Emotional abuse: ----+--, p=0.0062* **Emotional neglect: -------, p=1.5e-08*** Emotional neglect smoking: -------, p=1.6e-08* |
|  |  | chr3: 87138203-87138701 | 6 |  | ++++++ | 5.11E-06 | 0.00464 | ACE score: ++++++, p=0.068 Parent mentally ill: +++-++, p=0.028* Parent physically ill: +++--+, p=0.72 Parent died: -+++++, p=0.94 Parents separated: ------, p=0.5 Maternal bond: ++++++, p=0.8 Child illness: -+++++, p=0.96 **Child maltreatment: ++++++, p=0.0047*** | ACE score: ++++++, p=0.004* Parent mentally ill: ---+--, p=0.8 Parent physically ill: -+++++, p=0.019* Parent died: -+++-+, p=0.5 Parents separated: --++--, p=0.62 Maternal bond: ++++++, p=0.5 Child illness: ++++++, p=0.0088* Child maltreatment: ++++++, p=0.001* Physical abuse: ---++-, p=0.42 Physical neglect: ---+++, p=0.2 Sexual abuse: -+-+++, p=0.86 Emotional abuse: ---++-, p=0.41 **Emotional neglect: ++++++, p=5.1e-06*** Emotional neglect smoking: ++++++, p=4.1e-05* |
|  |  | chr11: 67417958-67418406 | 13 | *ACY3* | ------------- | 2.15E-10 | 2.17E-07 | ACE score: +++++--++-+-+, p=0.98 Parent mentally ill: ++++++++++++-, p=0.097 Parent physically ill: +-+---------+, p=0.73 Parent died: -+-+-++-----+, p=0.95 Parents separated: --+++-+++++-+, p=0.95 Maternal bond: +-++---+++--+, p=0.97 Child illness: +++----+----+, p=0.96 Child maltreatment: ++++++++++++-, p=0.0039* | ACE score: -------------, p=4.4e-05* Parent mentally ill: ++--+-+---++-, p=0.97 Parent physically ill: --+++++--+-+-, p=0.93 Parent died: -+++++++++++-, p=0.14 Parents separated: ---+----+--+-, p=0.78 Maternal bond: -------------, p=0.026* Child illness: +++++-+---+++, p=0.99 Child maltreatment: -------------, p=6e-13* Physical abuse: -------------, p=0.0021* Physical neglect: -+-----+-----, p=0.83 Sexual abuse: ----------+--, p=0.27 Emotional abuse: -------------, p=7.6e-08* **Emotional neglect: -------------, p=2.1e-10*** Emotional abuse smoking: -------------, p=2e-07*  Emotional neglect smoking: -------------, p=3.9e-08* |
|  |  | chr10: 47083283-47083633 | 9 | *PPYR1* | ++++++++- | 1.62E-06 | 0.002098 | ACE score: -------+-, p=0.4 Parent mentally ill: ----+++++, p=0.73 Parent physically ill: +----+---, p=0.47 Parent died: ---------, p=0.094 Parents separated: -++------, p=0.38 Maternal bond: -----+-++, p=0.79 Child illness: -++++-+++, p=0.62 Child maltreatment: --+--++++, p=0.77 | ACE score: +++++++--, p=0.29 Parent mentally ill: +-+--+---, p=0.77 Parent physically ill: +-+++++++, p=0.11 Parent died: +-++-----, p=0.7 Parents separated: ++++-----, p=0.76 Maternal bond: +----+---, p=0.47 Child illness: +-------+, p=0.093 Child maltreatment: ++++++++-, p=0.0017* Physical abuse: -++++----, p=0.36 Physical neglect: ----+----, p=0.61 Sexual abuse: +++-+---+, p=0.97 Emotional abuse: -++++-++-, p=0.73 **Emotional neglect: ++++++++-, p=1.6e-06*** Emotional neglect smoking: ++++++++-, p=1.5e-06* |
|  |  | chr2: 121338498-121338608 | 3 |  | --- | 4.67E-06 | 0.01909 | ACE score: ---, p=0.14 Parent mentally ill: +--, p=0.43 Parent physically ill: ---, p=0.099 Parent died: ---, p=0.55 Parents separated: ---, p=0.5 Maternal bond: ---, p=0.32 Child illness: +++, p=0.23 Child maltreatment: +++, p=0.73 | ACE score: ---, p=0.0041* Parent mentally ill: ---, p=0.023* Parent physically ill: +++, p=0.27 Parent died: +++, p=0.015* Parents separated: ++-, p=0.68 Maternal bond: ---, p=0.0035* Child illness: -++, p=0.6 Child maltreatment: ---, p=1.8e-05* Physical abuse: ---, p=0.13 Physical neglect: ---, p=0.011* Sexual abuse: ---, p=0.028* Emotional abuse: ---, p=0.00072* **Emotional neglect: ---, p=4.7e-06*** Emotional neglect smoking: ---, p=1.4e-05* |
|  |  |  |  |  |  |  |  |  |  |
| ALSPAC- Physical abuse | 8 | chr12: 132859608-132859952 | 4 | *GALNT9* | ++++ | 2.39E-06 | 0.003143 | ACE score: ++++, p=0.63 Parent mentally ill: ++++, p=0.36 Parent physically ill: ----, p=0.61 Parent died: ++++, p=3.2e-07* Parents separated: --++, p=0.8 Maternal bond: ++++, p=0.46 Child illness: ----, p=0.05 Child maltreatment: ++++, p=0.58 | ACE score: --++, p=0.92 Parent mentally ill: +++-, p=0.98 Parent physically ill: --++, p=0.47 Parent died: ----, p=0.46 Parents separated: +---, p=0.057 Maternal bond: ++++, p=0.35 Child illness: --++, p=0.85 Child maltreatment: -+--, p=0.67 **Physical abuse: ++++, p=2.4e-06*** Physical neglect: ++++, p=0.63 Sexual abuse: ----, p=1 Emotional abuse: --++, p=0.46 Emotional neglect: ----, p=0.34 Physical abuse smoking: ++++, p=0.00078* |
|  |  | chr1: 26503623-26504020 | 10 | *CNKSR1* | -+++++++++ | 5.70E-07 | 0.000651 | ACE score: ----------, p=0.018* Parent mentally ill: ++++++++++, p=0.49 Parent physically ill: -------+--, p=0.61 Parent died: +-+-------, p=0.43 Parents separated: ----------, p=0.37 Maternal bond: ----------, p=0.086 Child illness: +---------, p=0.93 Child maltreatment: +++-----+-, p=0.78 | ACE score: -++++++++-, p=0.66 Parent mentally ill: -+-+-+-+++, p=0.88 Parent physically ill: ++++++++++, p=1.5e-05* Parent died: +------+--, p=0.49 Parents separated: +---------, p=0.41 Maternal bond: ----+-----, p=0.45 Child illness: -+++++++++, p=2.1e-06* Child maltreatment: ----------, p=0.88 **Physical abuse: -+++++++++, p=5.7e-07*** Physical neglect: ----------, p=0.18 Sexual abuse: --++--++++, p=0.9 Emotional abuse: +--+--+---, p=0.9 Emotional neglect: --+-------, p=0.85 Physical abuse smoking: -+++++++++, p=1.5e-06* |
|  |  | chr1: 240656217-240656738 | 6 | *GREM2* | ------ | 5.39E-08 | 4.70E-05 | ACE score: ++++++, p=0.44 Parent mentally ill: ++---+, p=0.9 Parent physically ill: ++++++, p=0.78 Parent died: +++-++, p=0.93 Parents separated: ------, p=0.17 Maternal bond: ++++++, p=0.1 Child illness: +++++-, p=0.85 Child maltreatment: ------, p=0.38 | ACE score: -+----, p=0.84 Parent mentally ill: ------, p=0.075 Parent physically ill: +-+-+-, p=0.74 Parent died: +++++-, p=0.55 Parents separated: ---+++, p=0.63 Maternal bond: +-----, p=0.32 Child illness: +++--+, p=0.74 Child maltreatment: -++++-, p=0.79 **Physical abuse: ------, p=5.4e-08*** Physical neglect: ------, p=0.0055* Sexual abuse: ++----, p=0.55 Emotional abuse: -+----, p=0.61 Emotional neglect: -+++++, p=0.17 Physical abuse smoking: ------, p=6.4e-07* |
|  |  | chr1: 19600471-19600913 | 8 | *AKR7L* | ++++++++ | 4.69E-08 | 4.81E-05 | ACE score: +++++++-, p=0.67 Parent mentally ill: +++-+++-, p=0.8 Parent physically ill: +-+++-++, p=0.81 Parent died: +++++---, p=0.85 Parents separated: ++++++++, p=0.31 Maternal bond: --+++++-, p=0.91 Child illness: ++-+-++-, p=0.99 Child maltreatment: ------+-, p=0.6 | ACE score: +-++++-+, p=0.39 Parent mentally ill: --+++-++, p=0.56 Parent physically ill: +-+++--+, p=0.7 Parent died: --+-----, p=0.25 Parents separated: +-++++++, p=0.71 Maternal bond: --------, p=0.33 Child illness: +-++++++, p=0.16 Child maltreatment: +-++++++, p=0.48 **Physical abuse: ++++++++, p=4.7e-08*** Physical neglect: -+-++--+, p=0.85 Sexual abuse: ---+++++, p=0.79 Emotional abuse: ++++++++, p=0.1 Emotional neglect: +-++++-+, p=0.35 Physical abuse smoking: ++++++++, p=1.2e-07* |
|  |  | chr6: 28601269-28601520 | 13 |  | ------------- | 3.53E-08 | 6.37E-05 | ACE score: +++++++++++++, p=0.058 Parent mentally ill: +++--+---++++, p=1 Parent physically ill: --+----------, p=0.75 Parent died: +++-++++++++-, p=0.87 Parents separated: +++++++++++++, p=7.8e-05* Maternal bond: ++++++----+++, p=0.96 Child illness: +++++++++++++, p=4.6e-06* Child maltreatment: +-+----++---+, p=1 | ACE score: -------------, p=0.025* Parent mentally ill: -+-----------, p=0.24 Parent physically ill: -------++----, p=0.85 Parent died: -+-+-++++++++, p=0.7 Parents separated: -+-----------, p=0.074 Maternal bond: --+-------+-+, p=0.92 Child illness: --+-+--++----, p=0.82 Child maltreatment: --------+----, p=0.41 **Physical abuse: -------------, p=3.5e-08*** Physical neglect: +-+++++++++++, p=0.27 Sexual abuse: -------------, p=6.3e-05* Emotional abuse: -+--+++----++, p=0.96 Emotional neglect: --------+----, p=0.97 Physical abuse smoking: -------------, p=0.02* |
|  |  | chr7: 149569715-149570184 | 12 | *ATP6V0E2, LOC401431* | ++++++++++++ | 4.37E-07 | 0.000423 | ACE score: ++-+-+++++++, p=0.68 Parent mentally ill: +++--+++++++, p=0.56 Parent physically ill: +--++++-++-+, p=0.42 Parent died: -+++-++++++-, p=0.57 Parents separated: ++--+----+++, p=0.46 Maternal bond: ++++-+++-++-, p=0.55 Child illness: -+-++++++-++, p=0.58 Child maltreatment: ++-+-+++-++-, p=0.29 | ACE score: ---++++-+---, p=0.9 Parent mentally ill: --------+--+, p=0.4 Parent physically ill: ++-----+++-+, p=0.57 Parent died: -++++-+-++++, p=0.83 Parents separated: --+---+-+++-, p=0.9 Maternal bond: ---++-++-++-, p=0.51 Child illness: ++-++-+-++-+, p=0.98 Child maltreatment: +--++++---+-, p=0.93 **Physical abuse: ++++++++++++, p=4.4e-07*** Physical neglect: -+---++++--+, p=0.85 Sexual abuse: +---+---+---, p=0.63 Emotional abuse: --+-++-----+, p=0.76 Emotional neglect: ---++++-----, p=0.68 Physical abuse smoking: ++++++++++++, p=1.4e-06* |
|  |  | chr21: 47294981-47295376 | 3 | *PCBP3* | +++ | 1.12E-07 | 0.000129 | ACE score: +++, p=0.26 Parent mentally ill: +--, p=0.7 Parent physically ill: +++, p=0.68 Parent died: +--, p=0.74 Parents separated: +++, p=0.09 Maternal bond: +++, p=0.29 Child illness: ---, p=0.16 Child maltreatment: -++, p=0.33 | ACE score: +++, p=0.003* Parent mentally ill: +++, p=0.07 Parent physically ill: +++, p=0.017* Parent died: +++, p=0.18 Parents separated: +--, p=0.12 Maternal bond: +++, p=0.21 Child illness: +++, p=0.11 Child maltreatment: +++, p=0.011* **Physical abuse: +++, p=1.1e-07*** Physical neglect: --+, p=1 Sexual abuse: +++, p=0.97 Emotional abuse: +++, p=0.028* Emotional neglect: +++, p=0.17 Physical abuse smoking: +++, p=0.00032* |
|  |  | chr11: 36422377-36422616 | 5 | *PRR5L* | +++++ | 2.31E-06 | 0.00438 | ACE score: ++-+-, p=0.48 Parent mentally ill: ---+-, p=0.57 Parent physically ill: -----, p=0.45 Parent died: --++-, p=0.58 Parents separated: +++-+, p=0.89 Maternal bond: +++++, p=0.0022* Child illness: ----+, p=0.15 Child maltreatment: +---+, p=0.63 | ACE score: +++--, p=0.98 Parent mentally ill: --++-, p=0.51 Parent physically ill: ++-++, p=0.073 Parent died: +++-+, p=0.62 Parents separated: +----, p=0.67 Maternal bond: -++--, p=0.22 Child illness: +-+--, p=0.71 Child maltreatment: ++-+-, p=0.81 **Physical abuse: +++++, p=2.3e-06*** Physical neglect: +++++, p=0.67 Sexual abuse: ++-++, p=0.59 Emotional abuse: ++-++, p=0.76 Emotional neglect: ++-+-, p=0.86 Physical abuse smoking: +++++, p=5.8e-06* |
| ALSPAC- Physical neglect | 2 | chr6: 31505493-31505497 | 2 | *BAT1, SNORD117* | -- | 1.97E-07 | 0.02205 | ACE score: +-, p=0.54 Parent mentally ill: +-, p=0.38 Parent physically ill: ++, p=0.57 Parent died: -+, p=0.92 Parents separated: ++, p=0.035* Maternal bond: +-, p=0.84 Child illness: --, p=0.01* Child maltreatment: +-, p=0.45 | ACE score: --, p=0.24 Parent mentally ill: --, p=0.7 Parent physically ill: -+, p=0.79 Parent died: --, p=0.63 Parents separated: -+, p=0.17 Maternal bond: +-, p=0.72 Child illness: --, p=0.22 Child maltreatment: --, p=0.22 Physical abuse: --, p=0.017* **Physical neglect: --, p=2e-07*** Sexual abuse: --, p=0.61 Emotional abuse: -+, p=0.89 Emotional neglect: --, p=0.14 Physical neglect smoking: --, p=7.9e-05* |
|  |  | chr1: 27683139-27683502 | 5 | *MAP3K6* | +++++ | 5.86E-09 | 7.32E-06 | ACE score: ++---, p=0.43 Parent mentally ill: +++++, p=0.23 Parent physically ill: -----, p=0.023* Parent died: ++---, p=0.39 Parents separated: -----, p=0.3 Maternal bond: +++++, p=0.24 Child illness: +++-+, p=0.21 Child maltreatment: +----, p=0.067 | ACE score: -----, p=0.012* Parent mentally ill: +++++, p=0.83 Parent physically ill: -++-+, p=0.93 Parent died: ----+, p=0.54 Parents separated: -----, p=6e-04* Maternal bond: ----+, p=0.6 Child illness: -----, p=0.36 Child maltreatment: -----, p=0.0035* Physical abuse: -----, p=0.058 **Physical neglect: +++++, p=5.9e-09*** Sexual abuse: +-+++, p=0.95 Emotional abuse: ----+, p=0.85 Emotional neglect: -----, p=0.0013* Physical neglect smoking: +++++, p=8.3e-07* |
| ALSPAC- Sexual abuse | 1 | chr8: 112482677-112482696 | 2 |  | -- | 2.07E-07 | 0.004926 | ACE score: ++, p=0.56 ACEbin: ++, p=0.39 Parent mentally ill: +-, p=0.95 Parent physically ill: ++, p=0.96 Parent died: --, p=0.2 Parents separated: ++, p=0.77 Maternal bond: -+, p=0.97 Child illness: ++, p=0.07 Child maltreatment: ++, p=0.17 | ACE score: --, p=0.5 Parent mentally ill: --, p=0.36 Parent physically ill: ++, p=0.92 Parent died: +-, p=0.83 Parents separated: ++, p=0.96 Maternal bond: -+, p=0.96 Child illness: ++, p=0.27 Child maltreatment: --, p=0.0019* Physical abuse: ++, p=0.66 Physical neglect: --, p=0.38 **Sexual abuse: --, p=2.1e-07*** Emotional abuse: -+, p=0.83 Emotional neglect: --, p=0.019* Sexual abuse smoking: --, p=1.8e-07* |

*The first two columns indicate how many of the DMRs survive multiple testing correction (Šidák corrected p-value <0.05) in the original analysis, while the last eight columns give information on these replicated DMRs.*

** in the last two columns indicates a nominal significant association (p-value region <0.05) between the DMR and one of the childhood adversities in the respective cohort, NSHD or ALSPAC.*

***Supplementary Table 9*** *Lookup of the nine DMRs that replicated across cohorts in the antenatal ALSPAC DNA methylation dataset (n=769). The antenatal time point predates (approximately 17 years) the middle age ALSPAC sample used in the main analysis.*

| **ACE** | **DMR** | **Nr CpGs** | **Gene** | **ALSPAC middle age** (Direction^1^, p-value) | **ALSPAC antenatal** (Direction^1^, p-value) |
| --- | --- | --- | --- | --- | --- |
| ACE count score | chr8: 145654565-145654855 | 5 | *VPS28, TONSL* | -----  p=7.5e-08* | -----  p=1.2e-06* |
| Parent mentally ill | chr12: 14720726-14721289 | 10 | *PLBD1* | -++-++++++  p=0.0058 | -++++++---  p=0.27* |
| Parent mentally ill | chr1: 3104999-3105327 | 5 | *PRDM16* | ----- p=0.001 | ----+ p=0.13* |
| Parent physically ill | chr15: 81426347-81426670 | 9 | *C15orf26* | +++++++++ p=0.011 | +++++++++  p=2.435e-07* |
| Parent physically ill | chr22: 27834439-27834630 | 3 | n/a | ---  p=6.9e-06* | ---  p= 0.04 |
| Parent physically ill | chr8: 144120335-144120707 | 7 | *C8orf31* | +++++++  p=4.4e-06* | +++++++  p=0.001 |
| Parent died | chr15: 40364524-40364863 | 3 | n/a | +++  p=6.3e-07* | +++  p=0.74 |
| Parent died | chr7: 24323261-24323940 | 9 | *NPY* | +++++++++  p=9.2e-07* | +++++-+++  p=7.76e-05 |
| Parent died | chr2: 18766018-18766295 | 4 | *NT5C1B* | ---+  p=9.8e-06* | ---+  p=0.12 |

*^1^ Direction of effect for each individual CpG that is part of the DMR was derived from the regression coefficient in the epigenome wide analysis for individual CpGs.*

*^2^ If the DMR additionally replicated for another ACE (same direction of effect and StoufferLiptak-Kechris corrected P-value<0.05 in both ALSPAC and NSHD), the additional ACE is mentioned in this column.
* DMR passed Šidák correction for multiple testing in comb-p*

***
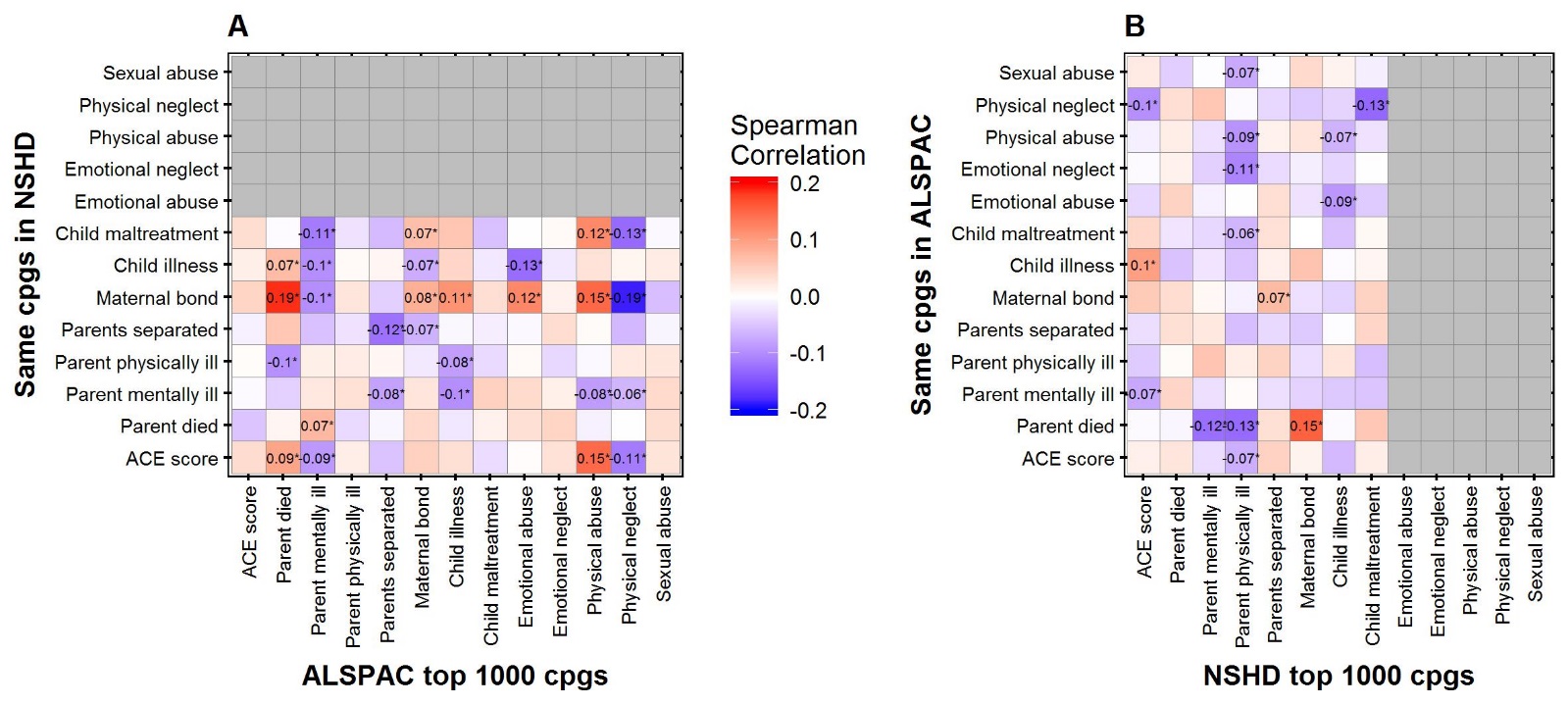
***

**Supplementary Figure 8** *Two heatmaps depicting the correlation of the coefficients of the top 1000 CpGs in ALSPAC (panel A) or NSHD (panel B) across cohorts. A higher positive correlation indicates the top 1000 CpGs have a similar effect size estimate in the other cohort. If there was significant correlation (p<0.05 in a spearman correlation), the correlation coefficient is given. The grey plotting area is due to the absence of specific childhood maltreatment measures in NSHD.*

*
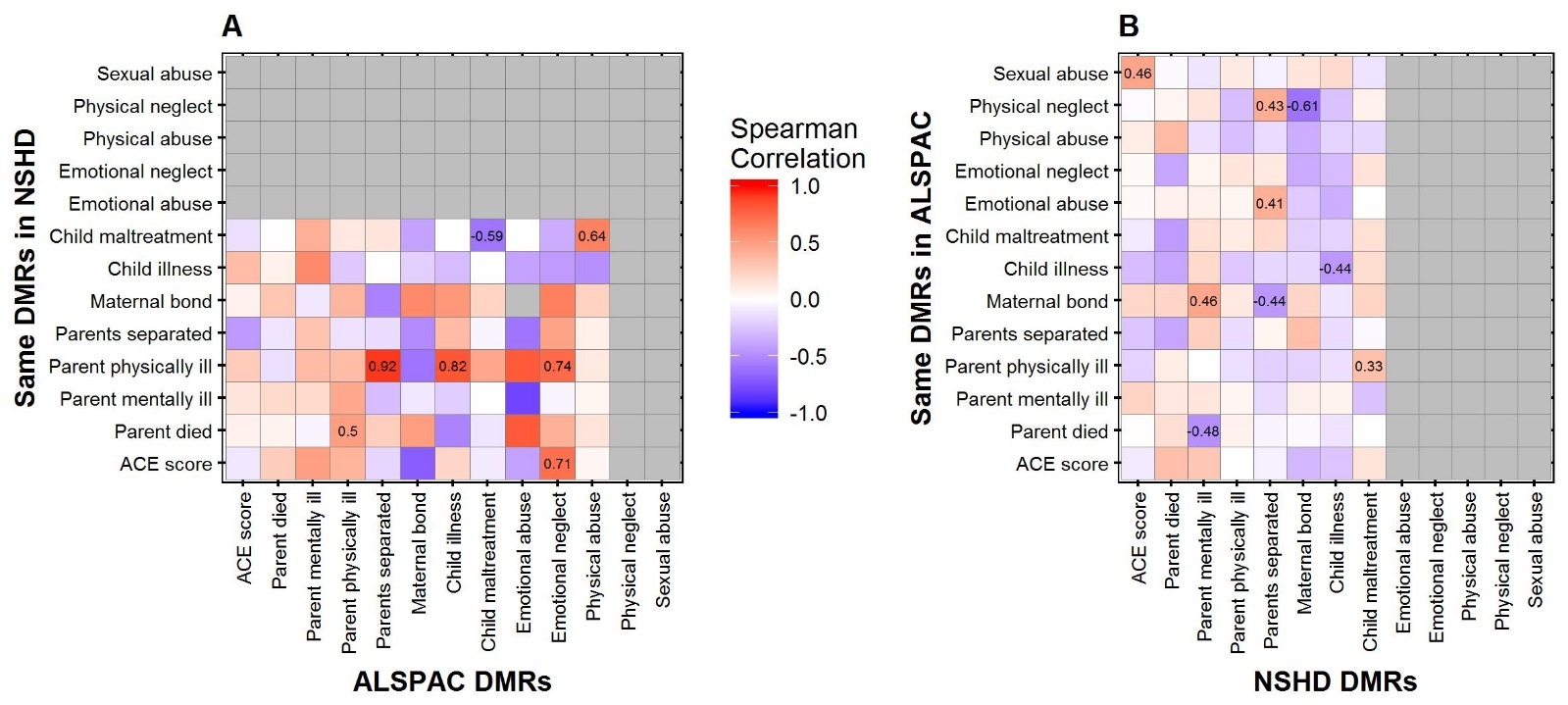
*

**Supplementary Figure 9** *Two heatmaps depicting the correlation of the average regression coefficients of the CpGs in ALSPAC (panel A) or NSHD (panel B) DMRs across cohorts. A higher positive correlation indicates the CpGs that are part of the DMR have a similar effect size estimate in the other cohort. If there was significant correlation (p<0.05 in a spearman correlation), the correlation coefficient is given. The grey plotting area is due to the absence of specific childhood maltreatment measures in NSHD.*

***
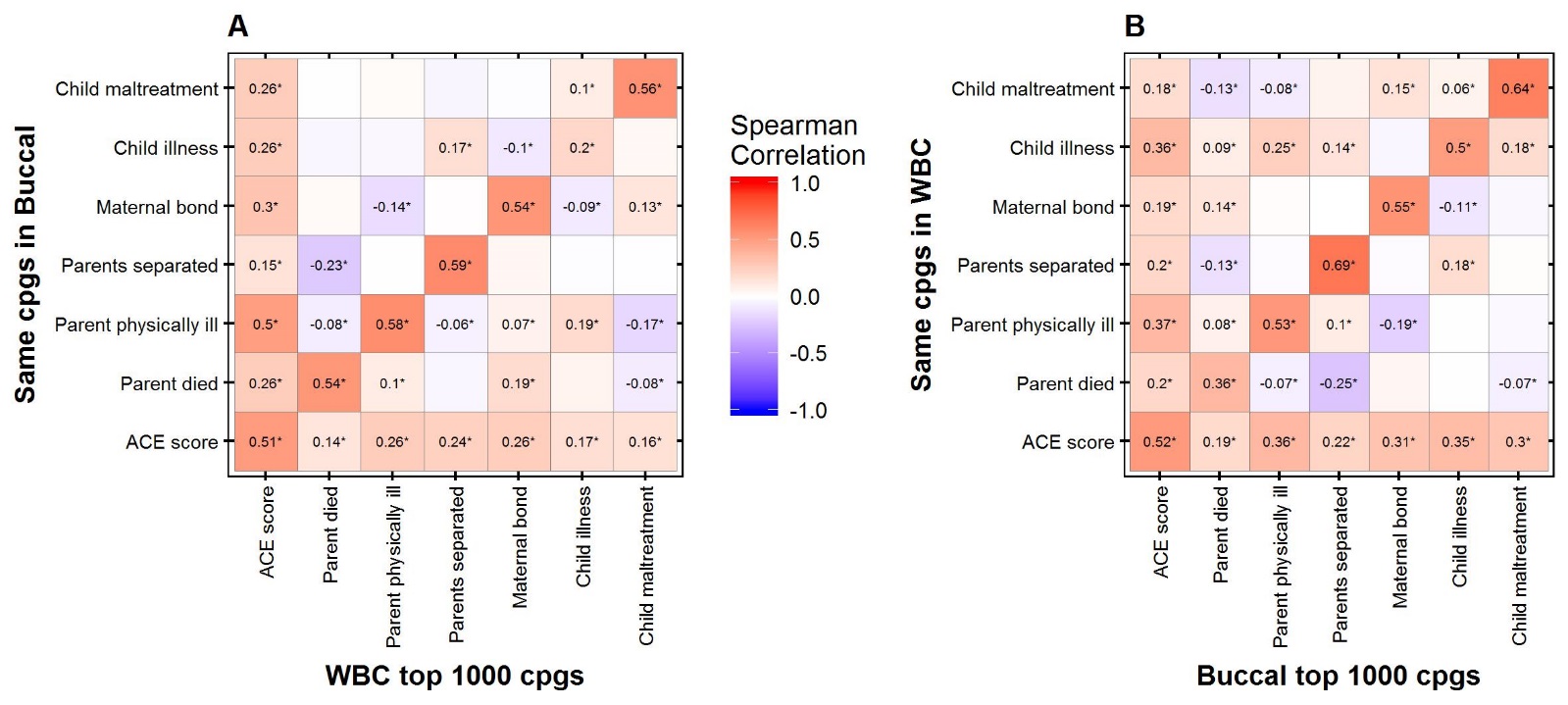
***

**Supplementary Figure 10** *Two heatmaps depicting the correlation of the coefficients of the top 1000 CpGs in blood (panel A) or buccal (panel B) tissue. A higher positive correlation indicates the top 1000 CpGs have a similar effect size estimate in the other tissue type. If there was significant correlation (p<0.05 in a spearman correlation), the correlation coefficient is given.*

*
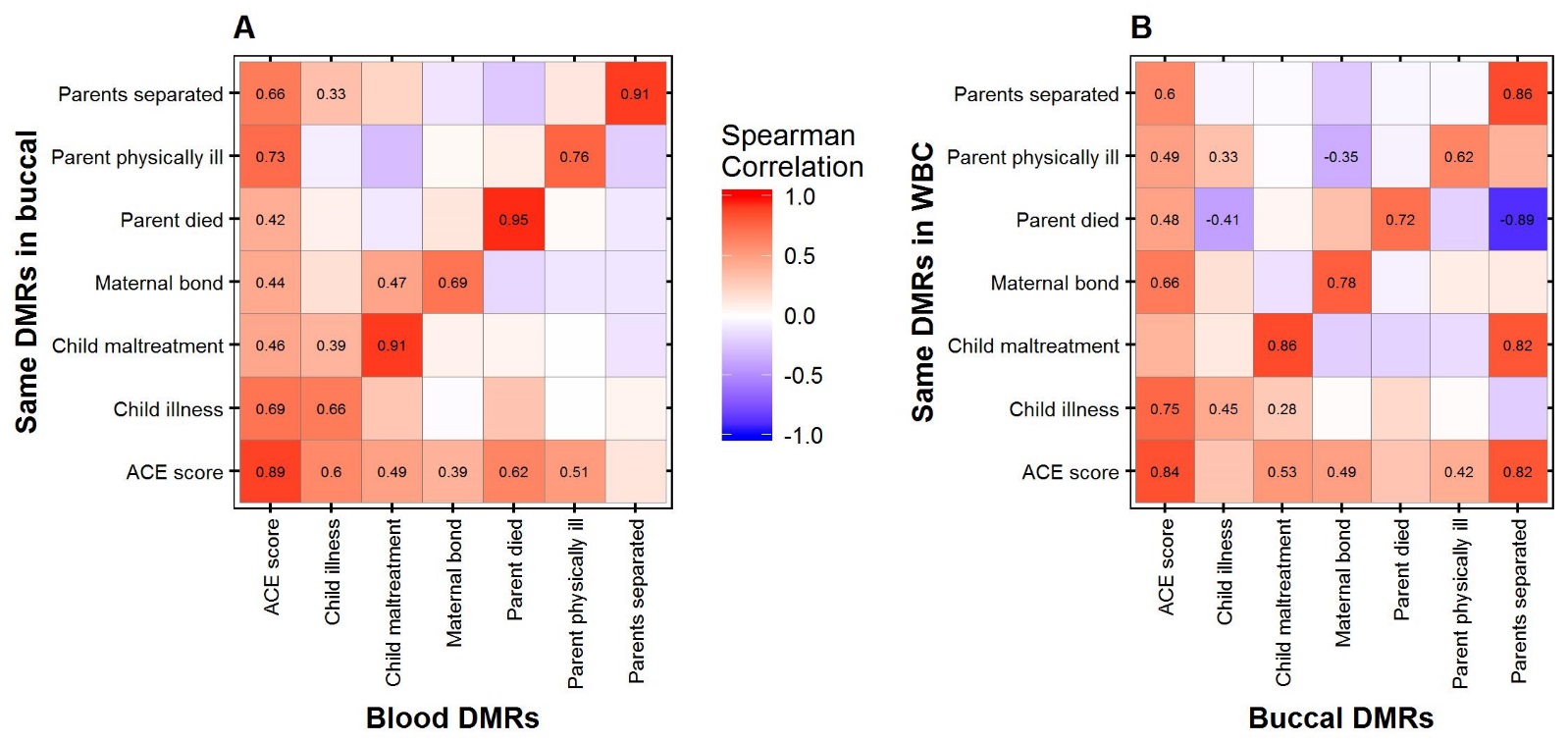
*

**Supplementary Figure 11** *Two heatmaps depicting the correlation of the average regression coefficients of the CpGs in blood (panel A) or buccal (panel B) DMRs across tissues. A higher positive correlation indicates the CpGs that are part of the DMR have a similar effect size estimate in the other tissue type. If there was significant correlation (p<0.05 in a spearman correlation), the correlation coefficient is given.*
